# Crystal structure determination and Hirshfeld surface analysis of *N*-acetyl-*N*-3-meth­oxy­phenyl and *N*-(2,5-di­meth­oxy­phen­yl)-*N*-phenyl­sulfonyl derivatives of *N*-[1-(phenyl­sulfon­yl)-1*H*-indol-2-yl]methanamine

**DOI:** 10.1107/S2056989024006649

**Published:** 2024-07-09

**Authors:** S. Madhan, M. NizamMohideen, Vinayagam Pavunkumar, Arasambattu K. MohanaKrishnan

**Affiliations:** a Department of Physics The New College Chennai 600 014 University of Madras,Tamil Nadu India; b Department of Organic Chemistry University of Madras, Guindy Campus Chennai-600 025 Tamilnadu India; National Taras Shevchenko University of Kyiv, Ukraine

**Keywords:** crystal structure, 1*H*-indole, acetamide, phenyl­sulfonamide, hydrogen bonding, Hirshfeld surface analysis

## Abstract

Two new [1-(phenyl­sulfon­yl)-1*H*-indol-2-yl]methanamine derivatives, namely, *N*-(3-meth­oxy­phen­yl)-*N*-{[1-(phenyl­sulfon­yl)-1*H*-indol-2-yl]meth­yl}acetamide, C_24_H_22_N_2_O_4_S, (**I**), and *N*-(2,5-di­meth­oxy­phen­yl)-*N*-{[1-(phenyl­sulfon­yl)-1*H*-indol-2-yl]meth­yl}benzene­sulfonamide, C_29_H_26_N_2_O_6_S_2_, (**II**), reveal a nearly orthogonal orientation of their indole ring systems and sulfonyl-bound phenyl rings. The sulfonyl moieties adopt the anti-periplanar conformation. For both compounds, the crystal packing is dominated by C—H⋯O bonding [C⋯O = 3.312 (4)–3.788 (8) Å], with the structure of **II** exhibiting a larger number, but weaker bonds of this type. Slipped π–π inter­actions of anti­parallel indole systems are specific for **I**, whereas the structure of **II** delivers two kinds of C—H⋯π inter­actions at both axial sides of the indole moiety. These findings agree with the results of Hirshfeld surface analysis. The primary contributions to the surface areas are associated with the contacts involving H atoms. Although **II** manifests a larger fraction of the O⋯H/H⋯O contacts (25.8 *versus* 22.4%), most of them are relatively distal and agree with the corresponding van der Waals separations.

## Abstract 2

The crystal structures of two 1*H*-indole derivatives are described and the inter­molecular contacts in the crystals are assessed and analysed using Hirshfeld surface analysis and two-dimensional fingerprint plots.

## 1. Chemical context

Derivatives of indole exhibit anti­bacterial (Okabe & Adachi, 1998 ▸) and anti­tumour (Schollmeyer *et al.*, 1995 ▸) activities. In particular, 1-(phenyl­sulfon­yl)indoles are applicable to the synthesis of biologically active alkaloids, such as the anti­cancer alkaloid ellipticine, carbazoles, furo­indoles, pyrrolo­indoles, indolocarbazoles and their analogues, including pyridocarbazoles. Some of the phenyl­sulfonyl indole compounds have been shown to inhibit the HIV-1 RT enzyme *in vitro* and HTLVIIIb viral spread in MT-4 human T-lymphoid cells (Williams *et al.*, 1993 ▸). In such systems, the phenyl­sulfonyl moiety can act either as a protecting or an activating group (Jasinski *et al.*, 2010 ▸). Ring-substituted acetanilides are valuable synthetic inter­mediates (Gowda *et al.*, 2007 ▸) that are used as precursors for the preparation of many heterocyclic compounds (Wen *et al.*, 2006 ▸). The amide linkage [–NHC(O)–] is known for its importance in maintaining protein architectures and it has been utilized in the development of mol­ecular devices for a spectrum of purposes in organic chemistry (NizamMohideen, SubbiahPandi *et al.*, 2009 ▸; NizamMohideen *et al.*, 2009*a* ▸,*b* ▸). Benzene­sulfonamide derivatives exhibit anti­tumor (Yang *et al.*, 2002 ▸), anti-bacterial (Badr, 2008 ▸) and anti-fungal (Hanafy *et al.*, 2007 ▸) activities. Recognizing the importance of such compounds for biochemical applications and drug discovery and our ongoing research into the construction of indole derivatives have prompted us to investigate a series of corresponding meth­oxy­phenyl-substituted species. We report herein the crystal structure determination and Hirshfeld surface analysis of two new (1-(phenyl­sulfon­yl)-1*H*-indol-2-yl)methanamine derivatives: *N*-(3-meth­oxy­phen­yl)-*N*-{[1-(phenyl­sulfon­yl)-1*H*-indol-2-yl]meth­yl}acetamide (**I**) and *N*-(2,5-di­meth­oxy­phen­yl)-*N*-{[1-(phenyl­sulfon­yl)-1*H*-indol-2-yl]meth­yl}benzene­sulfonamide (**II**).
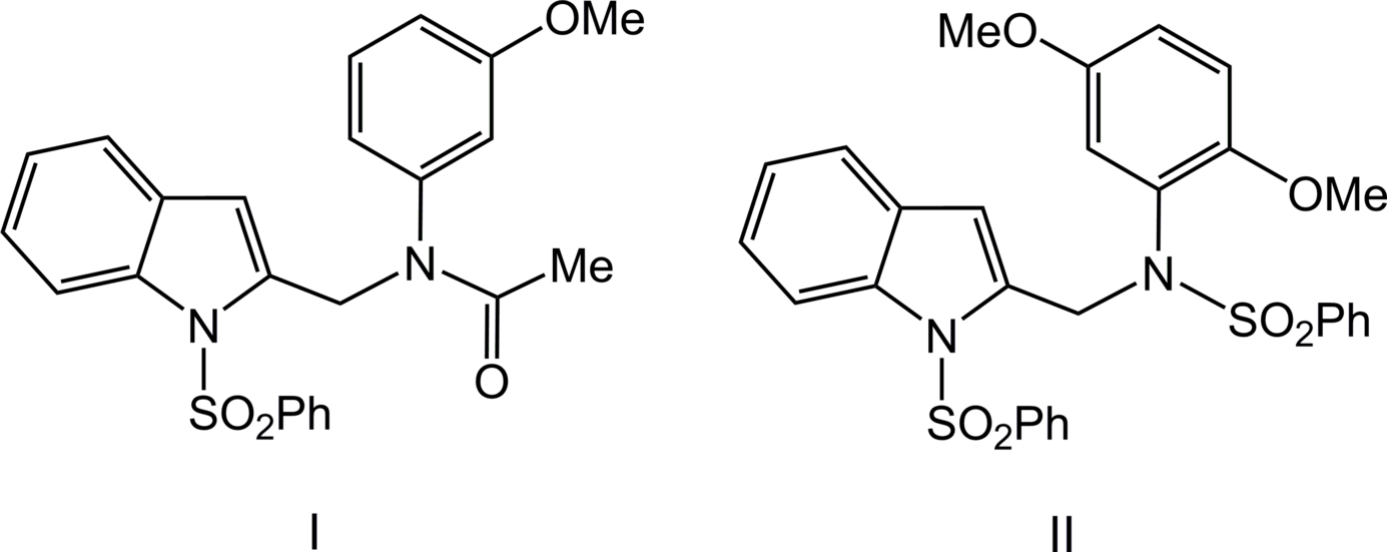


## 2. Structural commentary

**Figure 1:**
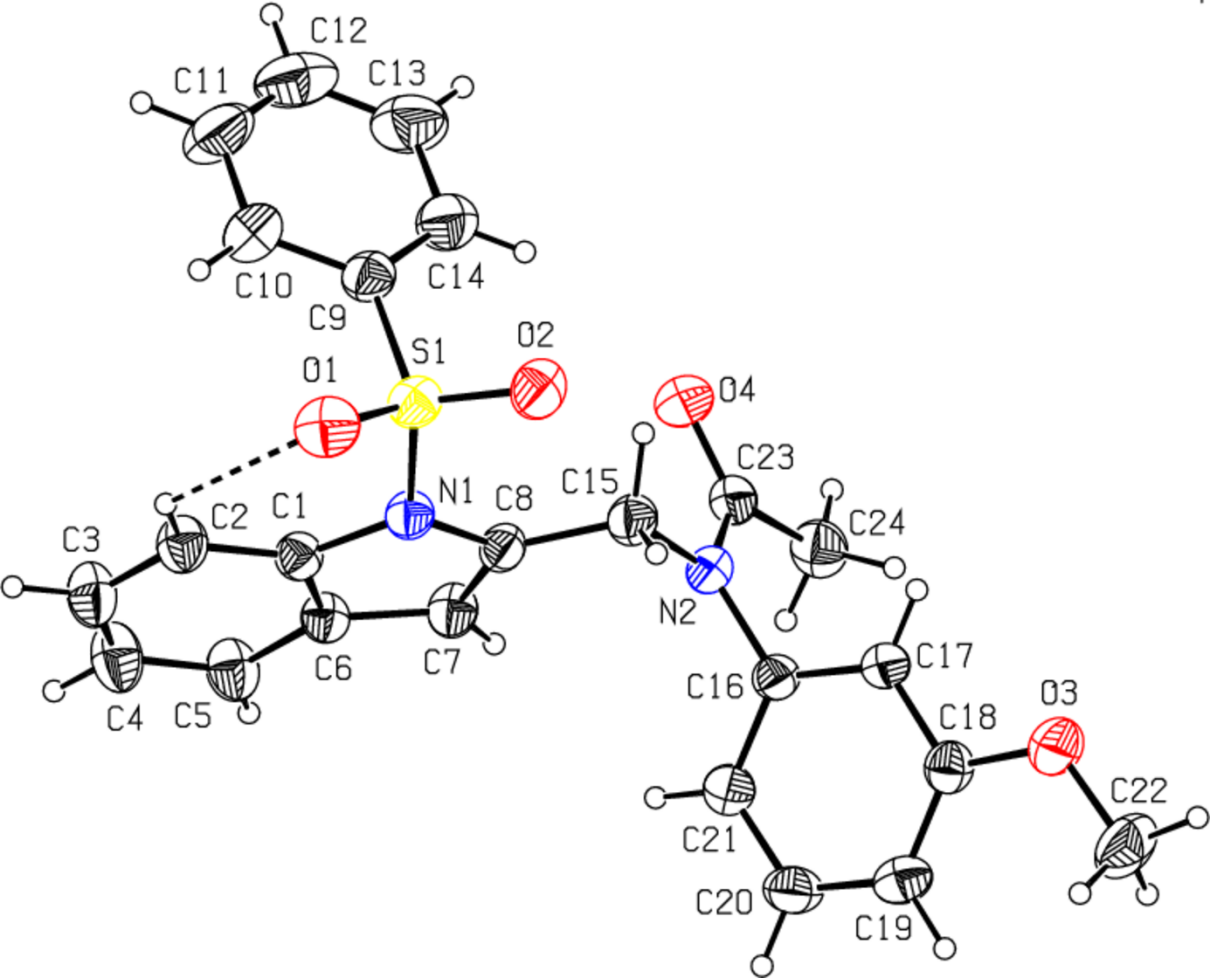
The mol­ecular structure of compound **I**, with atom labelling and displacement ellipsoids drawn at the 30% probability level. The dashed line indicates the intra­molecular hydrogen bond.

The mol­ecular structures of the title compounds, which differ in the substituents at the exocyclic nitro­gen atoms N2 [*N*-acetyl-*N*-3-meth­oxy­phenyl (**I**) and *N*-phenyl­sulfonyl-*N*-(2,5-di­meth­oxy­phen­yl) (**II**)], are illustrated in Figs. 1 ▸ and 2 ▸, respectively. In both compounds, the indole ring system (N1/C1–C8) is essentially planar, with maximum deviations from the corresponding mean planes of 0.027 (3) and 0.017 (5) Å observed for atoms C8 in **I** and C1 in **II**. The sulfonyl-bound phenyl rings (C9–C14) are almost orthogonal to the carrier indole ring systems (N1/C1–C8), with respective inter­planar angles of 83.9 (2)° for **I** and 83.5 (7)° for **II**. The meth­oxy-bound phenyl rings (C16–C21) in **I** and **II** are inclined to the indole frameworks, subtending dihedral angles of 66.31 (15) and 77.70 (9)°, respectively. In **I**, the planes of these outer phenyl rings (C9–C14 and C16–C21) subtend an angle of 59.8 (2)°, while in **II** they are nearly orthogonal [86.9 (9)°]. In the latter case, the dihedral angle between two sulfonyl-bound phenyl rings (C9–C14 and C24–C29) is 54.4 (2)°. The torsion angles O2—S1—N1—C1 and O1—S1—N1—C8 [177.3 (3) and −159.7 (3)° for **I** and −160.5 (5) and 164.0 (5)° for **II**, respectively] indicate the anti-periplanar conformation of the sulfonyl moiety. The geometric parameters of compounds **I** and **II** agree well with those reported for related structures [Madhan *et al.*, 2022 ▸, 2023*a* ▸,*b* ▸, 2024 ▸]. In both compounds, the tetra­hedral configuration around atom S1 is slightly distorted. The increase in the O2—S1—O1 angle [119.83 (17)° in **I** and 120.1 (3)° in **II**], with a simultaneous decrease in the N1—S1—C9 angle [104.54 (15)° in **I** and 105.9 (3)° in **II**] from the ideal tetra­hedral value (109.5°) are attributed to the Thorpe–Ingold effect (Bassindale, 1984 ▸). The widening of the angles may be due to the repulsive inter­action between the two short S=O bonds. In both compounds, as a result of the electron-withdrawing character of the phenyl­sulfonyl group, the N—C*sp*^2^ bond lengths [N1—C1 = 1.420 (4) in **I** and 1.429 (8) Å in **II** and N1—C8 = 1.427 (4) in **I** and 1.421 (7) Å in **II**] are longer than the mean value of 1.355 (14) Å for this bond (Allen *et al.*, 1987 ▸; Cambridge Structural Database (CSD), Version 5.37; Groom *et al.*, 2016 ▸). In both compounds, the sum of the bond angles around N1 [352.2 (2)° in **I** and 355.8 (2)° in **II**] indicate the *sp*^2^ hybridization (Beddoes *et al.*, 1986 ▸). In both compounds, the expansion of the *ipso* angles at atoms C1, C3 and C4, and the contraction of the apical angles at atoms C2, C5 and C6 is caused by fusion of the smaller pyrrole ring with the six-membered benzene ring and the strain is taken up by angular distortion rather than by bond-length distortion (Allen, 1981 ▸).

**Figure 2:**
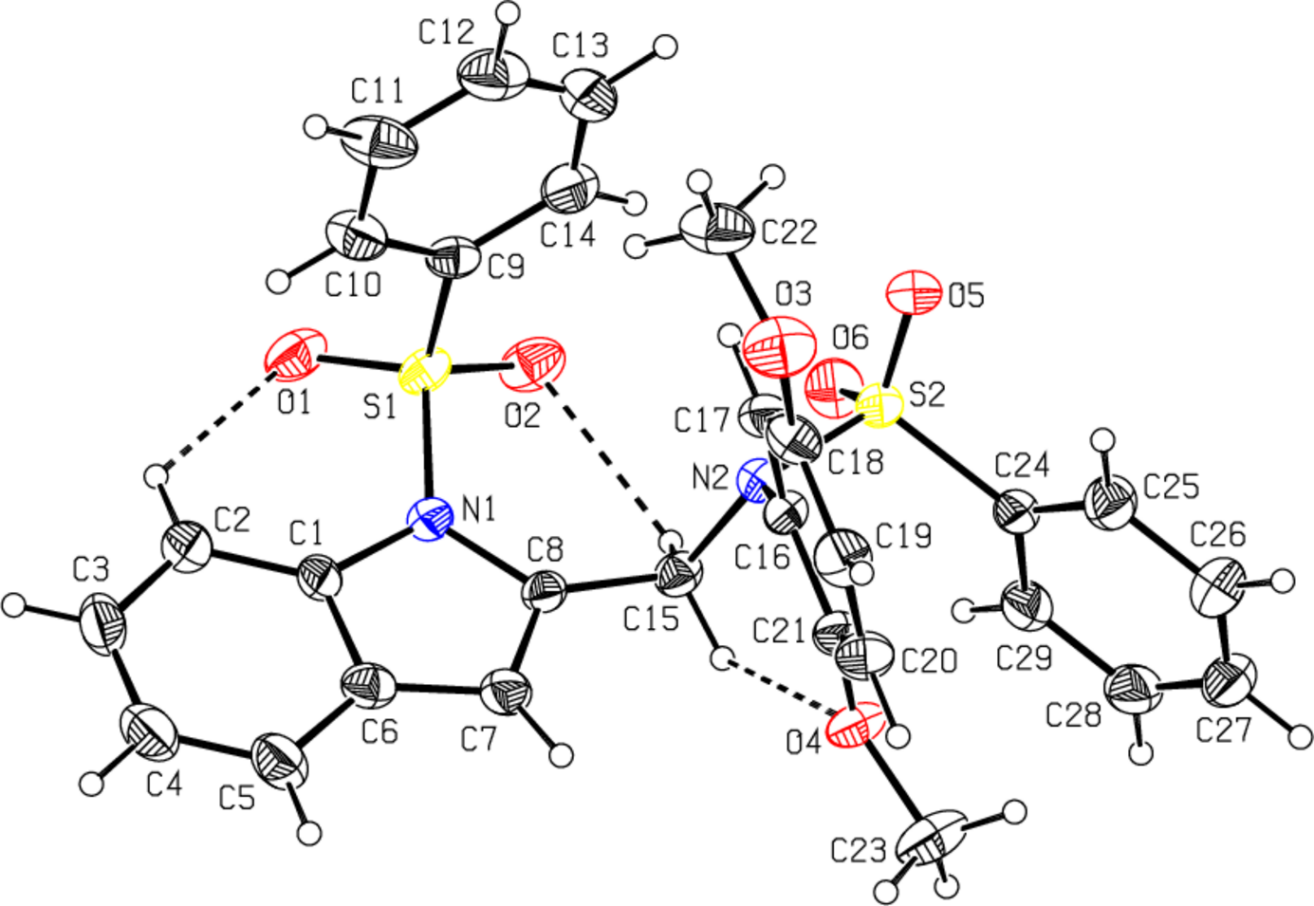
The mol­ecular structure of compound **II**, with atom labelling and displacement ellipsoids drawn at the 30% probability level. The dashed lines indicate the intra­molecular hydrogen bonds.

The mol­ecular conformation of compound **I** is stabilized by the weak intra­molecular hydrogen bond C2—H2⋯O1 [C2⋯O1 = 2.993 (5) Å] formed by the sulfone O atom, which generates an *S*(6) (N1/S1/O1/C1/C2/H2) ring motif (Fig. 1 ▸). A similar inter­action in compound **II** [C2⋯O1 = 2.886 (9) Å] is accompanied by two additional intra­molecular bonds involving methyl­ene donors and sulfone [C15⋯O2 = 2.948 (8) Å] and meth­oxy­phenyl [C15⋯O4 = 2.862 (8) Å] O atoms, which in total generate three *S*(6) ring motifs (N1/S1/O1/C1/C2/H2, N1/S1/O2/C8/C15/H15*B*) and N2/C16/C21/O4/C15/H15*A*), respectively (Fig. 2 ▸).

## 3. Supra­molecular features

**Figure 3:**
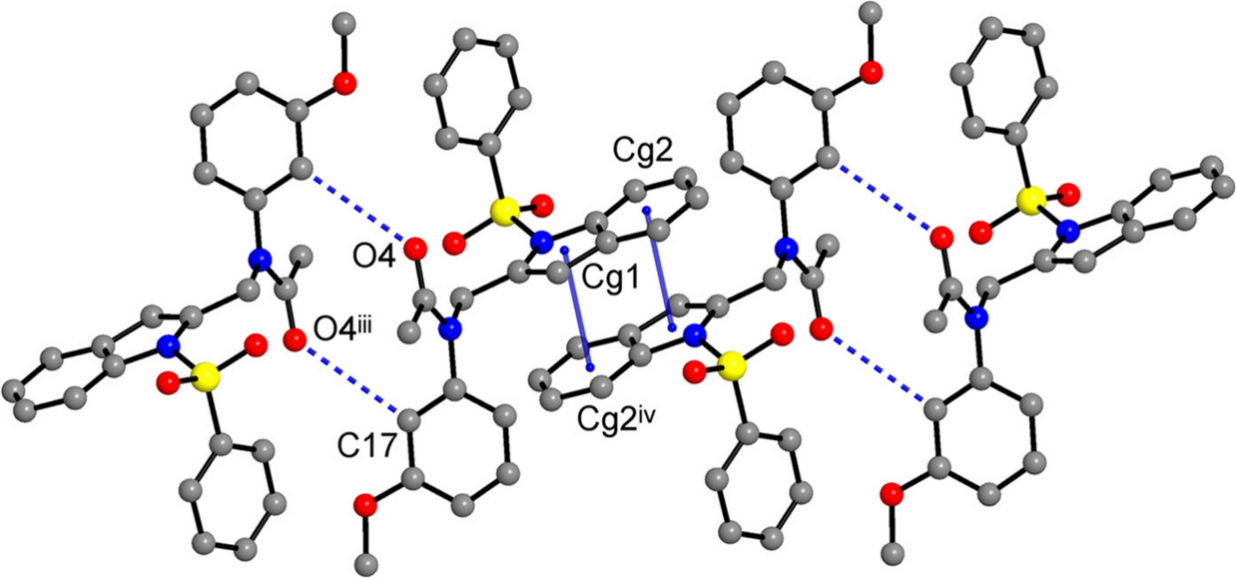
Fragment of non-covalent chain propagating along the *a*-axis direction in the structure of **I**, with the pairs of the inversion-related adjacent mol­ecules linked by double C—H⋯O bonds (dotted blue lines) and double π–π inter­actions (solid blue lines). [Symmetry codes: (iii) −*x* + 2, −*y* + 1, −*z* + 1; (iv) *x* + 1, −*y* + 1, −*z* + 1.]

**Figure 4:**
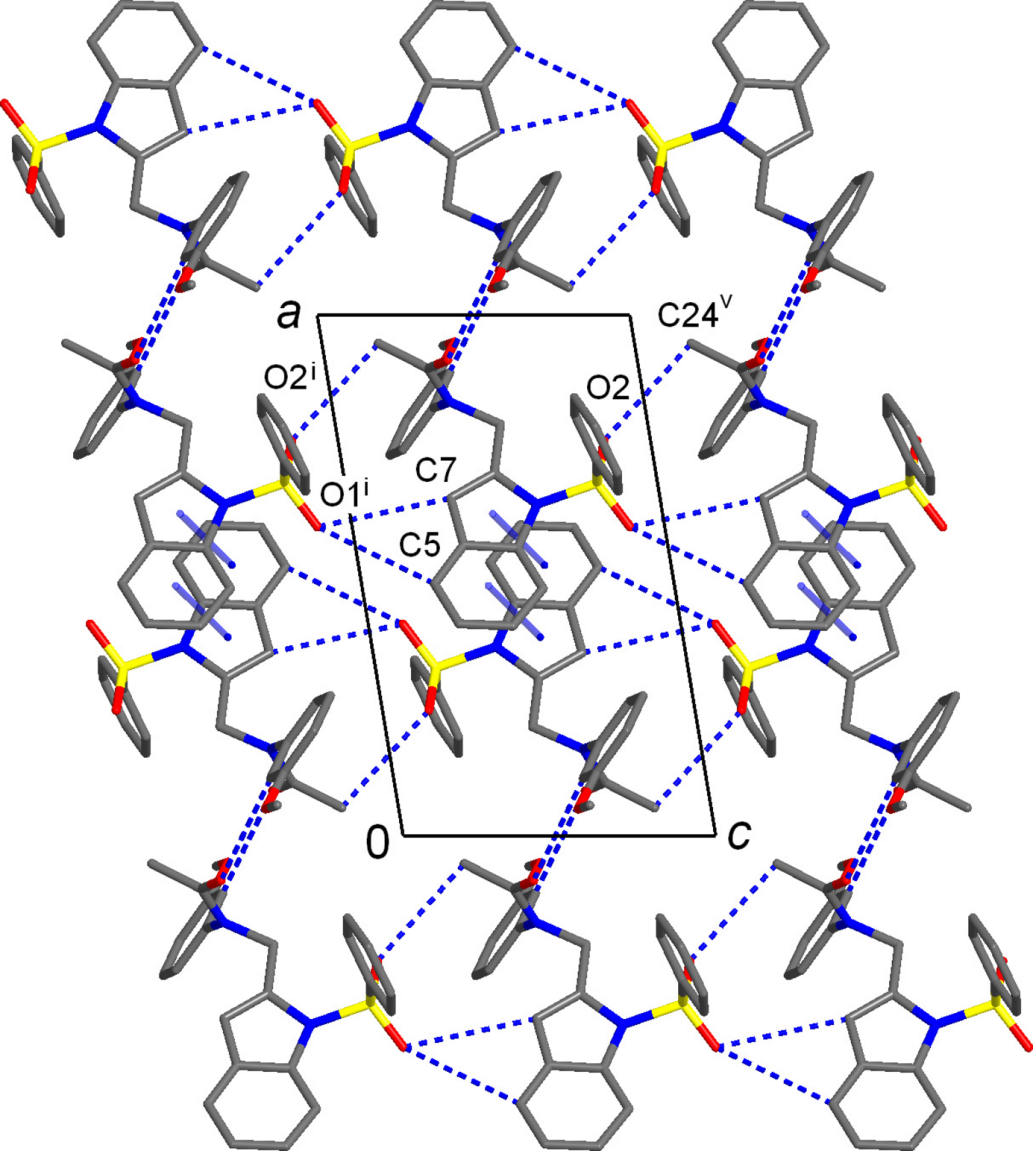
Projection of the structure of **I** on the *ac* plane, showing the layer assembled with C—H⋯O and π–π bonds. [Symmetry codes: (i) *x*, *y*, *z* − 1; (v) *x*, *y*, *z* + 1.]

With a lack of conventional hydrogen-bond donor functionality, the supra­molecular structures of both compounds are dominated by C—H⋯O bonding (Tables 1 ▸ and 2 ▸), whereas π–π inter­actions are specific for **I** and weaker C—H⋯π bonds are relevant for **II** only. In the crystal of **I**, the shortest hydrogen-bond contacts are observed for acetyl O-atom acceptors [C17⋯O4^iii^ = 3.312 (4) Å, symmetry code: (iii) −*x* + 2, −*y* + 1, −*z* + 1]. Such bonds assemble pairs of the mol­ecules into centrosymmetric dimers (Fig. 3 ▸) with a cyclic 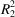
(12) (Bernstein *et al.*, 1995 ▸) ring motif. The dimers are further inter­connected into chains propagating along the *a*-direction through double π–π inter­actions of the indole ring systems (Fig. 3 ▸). The components of such stacks are related by inversion and therefore two indole systems are parallel, with inter­planar separation of 3.517 (4) Å. However, the overlap is only partial, as it is indicated by relatively large inter­centroid distances [*Cg*1⋯*Cg*2^iv^ = 3.801 (5) Å; *Cg*1 and *Cg*2 are the centroids of the N1/C1/C6–C8 and C1–C6 rings, respectively; symmetry code: (iv) −*x* + 1, −*y* + 1, −*z* + 1] and slippage angle of 22.3 (3)°. These parameters agree well with those for π–π inter­actions seen in the crystal structures of comparable 1-(phenyl­sulfon­yl)-1*H*-indole derivatives (Madhan *et al.*, 2024 ▸). Three C—H⋯O bonds with sulfone O-atom acceptors [C⋯O = 3.410 (5)–3.537 (4) Å; Table 1 ▸] are important for connection of the above chains into layers parallel to the *ac* plane (Fig. 4 ▸) and separated by 9.890 Å, which is half of the *b*-axis parameter of the unit cell. Only one C—H⋯O bond occurs between the layers, involving the sterically most accessible acetyl O-atom acceptor [C12⋯O4^ii^ = 3.527 (6) Å; symmetry code: (ii) *x*, −*y* + 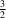, *z* + 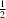]. No significant C—H⋯π inter­actions with C⋯centroid distances below 4 Å are observed in the structure.

**Figure 5:**
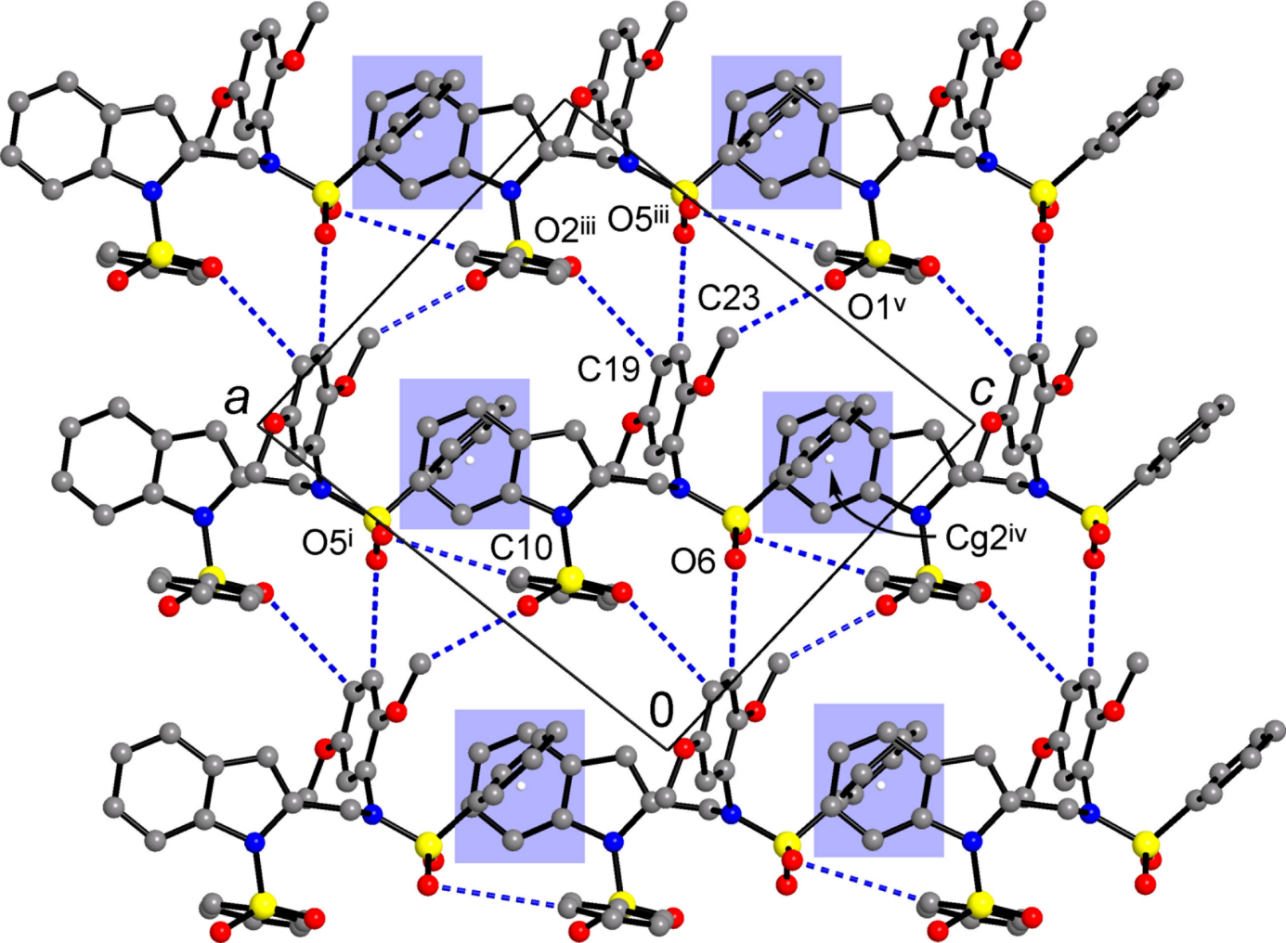
The non-covalent layer in the structure of **II**, viewed in a projection on the *ac* plane. Dotted blue lines represent CH⋯O bonds and short tetrel bonds C23⋯O1^v^, while blue areas indicate short C—H⋯π bonds with the sulfonyl-bound phenyl donors situated nearly orthogonal to the plane of the drawing. [Symmetry codes: (i) *x* + 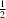, −*y* + 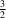, *z* − 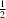; (iii) *x* + 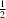, −*y* + 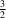, *z* + 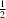; (iv) *x* − 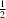, −*y* + 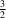, *z* + 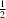; (v) *x*, *y*, *z* + 1.]

**Figure 6:**
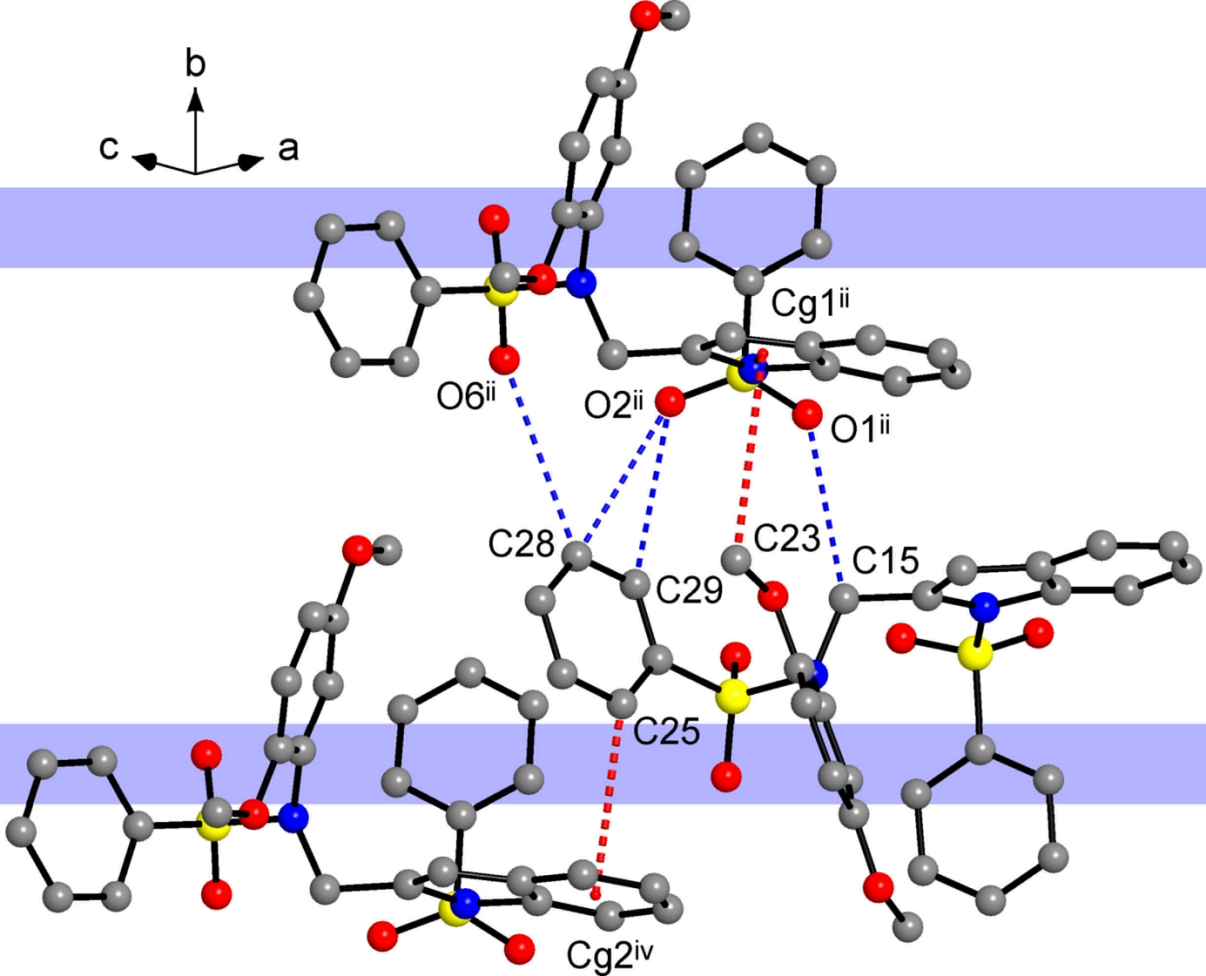
Set of inter­layer bonds in the structure of **II**, with the C—H⋯O and C—H⋯π bonds marked with dashed blue and red lines, respectively. Blue strips indicate two successive layers, which are nearly orthogonal to the plane of the drawing. [Symmetry codes: (ii) *x*, −*y* + 2, *z* + 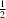; (iv) *x* − 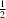, −*y* + 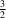, *z* + 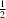.]

Similar non-covalent layers parallel to the *ac* plane are also seen in compound **II** (Fig. 5 ▸). However, the bonding pattern differs as π–π inter­actions are replaced by C—H⋯π inter­actions (on both axial sides of the indole system) and more extensive C—H⋯O bonding (Table 2 ▸). This is in line with increased number of hydrogen-bond donors and acceptors due to the incorporation of the additional phenyl­sulfonyl groups. The layers are sustained by a number of C—H⋯O inter­actions, which are relatively weak and distal [C⋯O = 3.503 (9)–3.788 (8)Å]. Significantly shorter contacts adopted by methyl groups are also present: C23⋯O1^v^ = 3.199 (7) Å; symmetry code: (v) *x*, *y*, *z* + 1. As a result of inappropriate angles at the H atoms, these contacts are not regarded as hydrogen bonds, rather representing a kind of tetrel inter­action CH_3_⋯O. A salient feature of the layer concerns C—H⋯π inter­actions involving the C1–C6 rings, which are appreciably short and directional [C25⋯*Cg*2^iv^ = 3.483 (5) Å; C25—H25⋯*Cg*2^iv^ = 147°; *Cg*2 is the C1–C6 ring centroid; symmetry code: (iv) *x* − 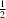, −*y* + 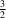, *z* + 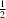]. The shortest inter­layer inter­actions represent C—H⋯O bonds with the most polarized methyl­ene donors [C15⋯O1^ii^ = 3.333 (8) Å; symmetry code: (ii) *x*, −*y* + 2, *z* + 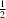], which act in synergy with a set of longer C—H⋯O (phen­yl) bonds and weak C—H⋯π bonds to the indole (N1/C1/C6–C8) acceptors (Fig. 6 ▸). In comparison with the structure of **I**, the much more extensive inter­actions in the present case result in a lower inter­layer spacing of 8.596 Å, which is a half of the *b*- axis parameter of the unit cell. This contributes to a slightly higher packing index of 68.1% *versus* 66.9% for **I**. However, in both the cases, the packing indices approach the lower limit of the 65–75% range expected for organic solids (Dunitz, 1995 ▸), suggesting relatively loose packing of these sterically strained mol­ecules.

## 4. Hirshfeld surface analysis

**Figure 7:**
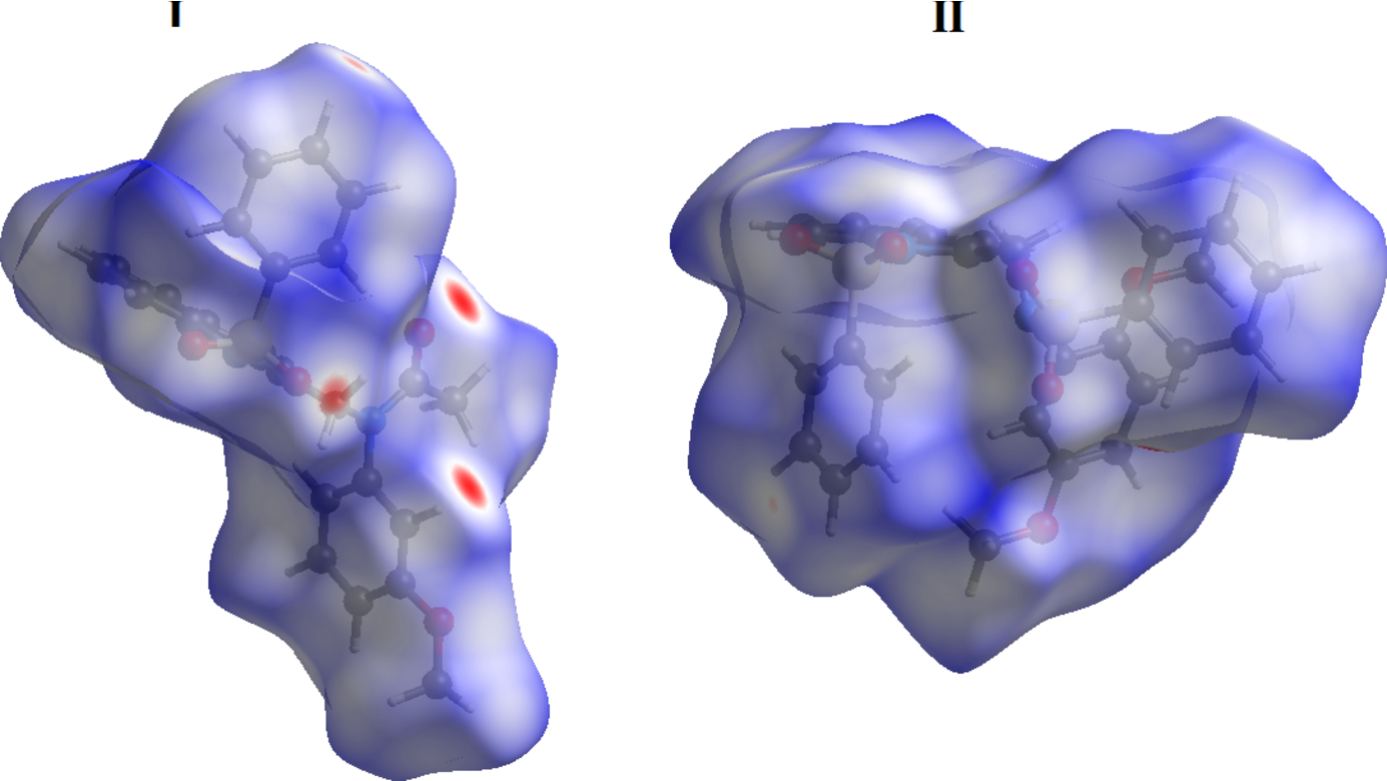
The Hirshfeld surfaces of compounds **I** and **II** mapped over *d_norm_*.

The Hirshfeld surface calculations and associated two-dimensional fingerprint plots for **I** and **II** were performed in accord with established procedures (Tan *et al.*, 2019 ▸) using *Crystal Explorer* (Spackman *et al.*, 2021 ▸) to determine the influence of weak inter­molecular inter­actions upon the mol­ecular packing in the absence of conventional hydrogen bonds. The Hirshfeld surfaces for two compounds mapped over *d*_norm_ using a fixed colour scale of −0.249 (red) to 1.450 a.u. (blue) for **I** and −0.096 (red) to 1.442 a.u. (blue) for **II** are shown in Fig. 7 ▸. One can note a relatively scarce landscape of short contacts that is particularly the case for **II**, which shows normal van der Waals separations only (denoted with several white regions on the surface). The few red spots present in the case **I** indicate inter­molecular contacts involved in weak hydrogen bonding.

**Figure 8:**
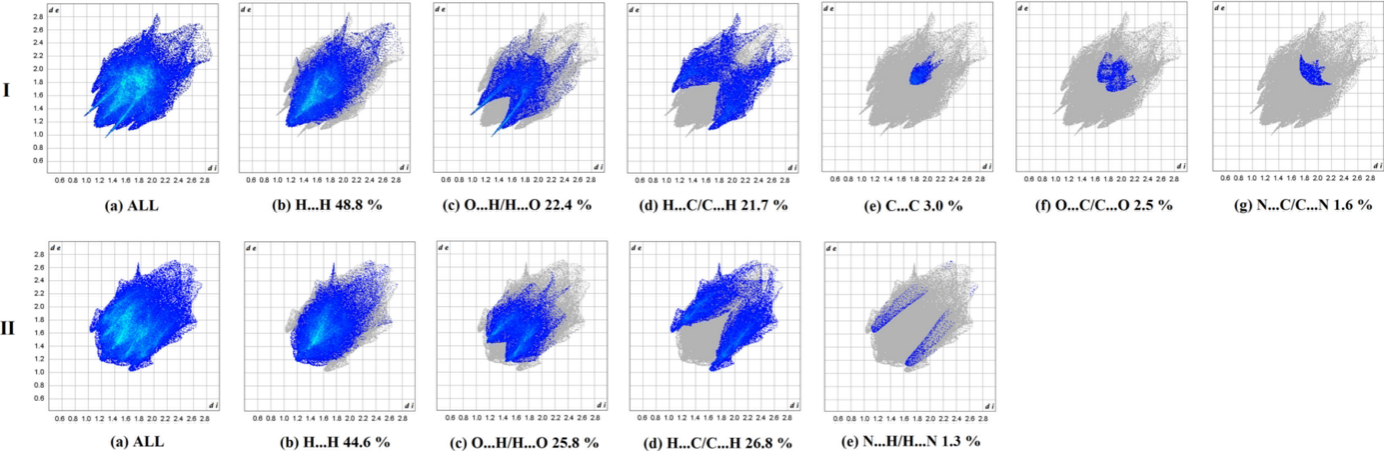
Two-dimensional fingerprint plots for **I** and **II** and those delineated into the principal contributions of H⋯H, C⋯H/H⋯C, O⋯H/H⋯O, N⋯H/H⋯N, C⋯C, O⋯C/C⋯O and N⋯C/C⋯N contacts. Other contributors account for less than 1.0% contacts to the surface areas.

The two-dimensional fingerprint plots (Parkin *et al.*, 2007 ▸) detailing the various inter­actions for the mol­ecules are shown in Fig. 8 ▸. For both compounds, the Hirshfeld surfaces suggest dominance of contacts with hydrogen atoms, accounting for over 85% of the contacts. Beyond the largest fractions of H⋯H contacts (48.8 and 44.6%), these short separations are overwhelmingly O⋯H/H⋯O and C⋯H/H⋯C, which contribute 22.4 and 21.7%, respectively, to the Hirshfeld surface in **I** and 25.8 and 26.8%, respectively, in **II**, respectively. The plots also illustrate the finding discussed above that the structure of **II** exhibits a larger number, but essentially weaker C—H⋯O bonds. Thus, for **I** the O⋯H/H⋯O plot represents pair of broad spikes pointing to the lower left, with the shortest contact being 2.35 Å, whereas in the case of **II** the diffuse and faintly discernible spikes are much shorter (O⋯H = 2.70 Å). The larger contribution of C⋯H/H⋯C contacts for **II** (Fig. 8 ▸) reflects the increased significance of C—H⋯π inter­actions for the crystal packing, in line with increased number of aromatic groups. The small fraction of N⋯H/H⋯N contacts (1.3%) is also a consequence of C—H⋯π bonding, namely with the pyrrole ring acceptor. An overlap between the parallel indole ring systems in **I**, due to the slipped π–π inter­actions, is clearly indicated by the plots for C⋯C, N⋯C/C⋯N and O⋯C/C⋯O (total contribution is 7.1%), in the form of the blue areas centered at *ca d*_e_ = *d*_i_ = 1.90 Å and with shortest contacts of 3.50 Å (Fig. 8 ▸). This weak bonding complements the above inter­actions involving H atoms. For both compounds, the H⋯H inter­molecular contacts predominate, followed by the C⋯H/H⋯C and O⋯H/H⋯O contacts. The Hirshfeld surface analysis confirms the importance of distal H-atom contacts (and contacts associated with the π–π inter­action for **I**) in establishing the packing.

## 5. Database survey

A search of the Cambridge Structural Database (Version 5.37; Groom *et al.*, 2016 ▸) indicated 123 compounds incorporating the phenyl­sulfonyl-1*H*-indole moiety. Of these, the most closely related examples are provided by structures of bromo-substituted 3-methyl-1-(phenyl­sulfon­yl)-1*H*-indole derivatives (JOMJII, JOMJAA and JOMJEE; Madhan *et al.*, 2024 ▸), ethyl 2-acet­oxy­methyl-1-phenyl­sulfonyl-1*H*-indole-3-carboxyl­ate (HUCQUS; Gunasekaran *et al.*, 2009 ▸), 3-iodo-2-methyl-1-phenyl­sulfonyl-1*H*-indole (ULESEK; Ramathilagam *et al.*, 2011 ▸) and 1-(2-bromo­methyl-1-phenyl­sulfonyl-1*H*-indol-3-yl)propan-1-one (CIQFEP; Umadevi *et al.*, 2013 ▸). In these structures, the sulfonyl-bound phenyl rings are almost orth­ogonal to the indole ring systems [the corresponding dihedral angles are in the range 73.35 (7)–89.91 (11)°], being comparable with those in the present two compounds.

## 6. Synthesis and crystallization

Compound **I**: 3-meth­oxy-*N*-{[1-(phenyl­sulfon­yl)-1*H*-indol-2-yl]meth­yl}aniline (0.100 g, 0.255 mmol) was dissolved in 5 ml of acetic anhydride and the reaction mixture was stirred for 8 h at 343 K. After completion of the reaction (monitored by TLC, *R*_f_ = 0.30, hexa­ne–ethyl acetate 80:20 *v*/*v*), the solution was poured into crushed ice (50 g), the solid formed was filtered, washed with 100 ml of water and dried over anhydrous CaCl_2_. Recrystallization of the crude product from diethyl ether (10 mL) afforded *N*-(3-meth­oxy­phen­yl)-*N*-{[1-(phenyl­sulfon­yl)-1*H*-indol-2-yl]meth­yl}acetamide as a colourless solid (84 mg, 76%), m.p. = 413–415 K. ^1^H NMR (300 MHz, CDCl_3_), δ, p.p.m.: 8.06 (*d*, *J* = 7.8 Hz, 1H), 7.74 (*d*, *J* = 7.8 Hz, 2H), 7.50–7.34 (*m*, 4H), 7.29–7.16 (*m*, 3H), 6.87–6.79 (*m*, 3H), 6.61 (*s*, 1H), 5.35 (*s*, 2H), 3.76 (*s*, 3H), 2.04 (*s*, 3H). ^13^C{^1^H} NMR (75 MHz, CDCl_3_), δ, p.p.m.: 170.7, 160.4, 144.2, 138.3, 137.1, 137.0, 133.7, 130.3, 129.5, 129.2, 126.3, 124.4, 123.7, 120.6, 119.7, 114.5, 113.4, 113.3, 110.4, 55.4, 48.2, 22.6.

Compound **II**: To a solution of 2-(bromo­meth­yl)-1-(phenyl­sulfon­yl)-1*H*-indole (0.710 g, 2.040 mmol) in CH_3_CN (10 ml), K_2_CO_3_ (0.422 g, 3.060 mmol) and *N*-(2,5-di­meth­oxy­phen­yl)benzene­sulfonamide (0.717 g, 2.448 mmol) were added and the mixture was stirred at room temperature for 12 h. After completion of the reaction (monitored by TLC, *R*_f_ = 0.60, hexane-ethyl acetate 80:20 *v*/*v*), the mixture was poured into crushed ice (50 g) containing 1 mL of concentrated HCl solution. The mixture was extracted with ethyl acetate (2 × 20 ml), the extracts were washed with water (2 × 20 ml) and dried over anhydrous Na_2_SO_4_. Removal of the solvent *in vacuo* followed by trituration of the crude product with 5 ml of methanol afforded *N*-(2,5-di­meth­oxy­phen­yl)-*N*-{[1-(phenyl­sulfon­yl)-1*H*-indol-2-yl]meth­yl}benzene­sulfonamide (0.802 g, 70%) as colourless solid, m.p. = 409–411 K. ^1^H NMR (300 MHz, CDCl_3_), δ, p.p.m.: 7.95 (*d*, *J* = 7.8 Hz, 1H), 7.66–7.57 (*m*, 4H), 7.53–7.46 (*m*, 1H), 7.43–7.35 (m, 4H), 7.32–7.24 (*m*, 2H), 7.21–7.08 (*m*, 2H), 7.00–6.90 *(m*, 2H), 6.73 (*dd*, *J*_1_ = 9.0 Hz, *J*_2_ = 2.7 Hz, 1H), 6.63–6.54 (*m*, 1H), 5.23 (*s*, 2H), 3.65 (*s*, 3H), 3.24 (*s*, 3H). ^13^C{^1^H} NMR (75 MHz, CDCl_3_), δ, p.p.m.: 153.1, 150.1, 139.6, 138.3, 138.1, 137.3, 133.7, 132.5, 129.7, 129.2, 128.5, 127.7, 127.2, 126.3, 124.4, 123.8, 120.9, 118.9, 114.9, 114.5, 112.1, 111.6, 55.8, 55.3, 49.1.

## 7. Refinement

Crystal data, data collection and structure refinement details are summarized in Table 3 ▸. All C-bound H atoms were positioned geometrically and constrained to ride on their parent atoms: C—H = 0.93–0.97 Å with *U*_iso_(H) = 1.5*U*_eq_(C-meth­yl) and 1.2*U*_eq_(C) for other H atoms.

## Supplementary Material

Crystal structure: contains datablock(s) global, I, II. DOI: 10.1107/S2056989024006649/nu2006sup1.cif

Structure factors: contains datablock(s) I. DOI: 10.1107/S2056989024006649/nu2006Isup2.hkl

Structure factors: contains datablock(s) II. DOI: 10.1107/S2056989024006649/nu2006IIsup3.hkl

Supporting information file. DOI: 10.1107/S2056989024006649/nu2006Isup4.cml

Supporting information file. DOI: 10.1107/S2056989024006649/nu2006IIsup5.cml

CCDC references: 2368308, 2368307

Additional supporting information:  crystallographic information; 3D view; checkCIF report

## Figures and Tables

**Table 1:** Hydrogen-bond geometry (Å, °) for **I**[Chem scheme1]

*D*—H⋯*A*	*D*—H	H⋯*A*	*D*⋯*A*	*D*—H⋯*A*
C2—H2⋯O1	0.93	2.41	2.993 (5)	120
C5—H5⋯O1^i^	0.93	2.75	3.530 (5)	143
C7—H7⋯O1^i^	0.93	2.81	3.537 (4)	135
C12—H12⋯O4^ii^	0.93	2.62	3.527 (6)	164
C13—H13⋯O3^iii^	0.93	2.69	3.591 (7)	164
C17—H17⋯O4^iii^	0.93	2.42	3.312 (4)	161
C24—H24*B*⋯O2^i^	0.96	2.49	3.410 (5)	160

Symmetry codes: (i) 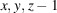; (ii) 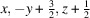; (iii) 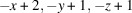.

**Table 2:** Hydrogen-bond geometry (Å, °) for **II**[Chem scheme1] *Cg*1 and *Cg*2 are the centroids of the N1/C1/C6–C8 and C1–C6 rings, respectively.

*D*—H⋯*A*	*D*—H	H⋯*A*	*D*⋯*A*	*D*—H⋯*A*
C2—H2⋯O1	0.93	2.30	2.886 (9)	121
C15—H15*A*⋯O4	0.97	2.23	2.862 (8)	122
C15—H15*B*⋯O2	0.97	2.34	2.948 (8)	120
C10—H10⋯O5^i^	0.93	2.93	3.719 (9)	144
C11—H11⋯O6^i^	0.93	2.85	3.723 (11)	156
C15—H15*A*⋯O1^ii^	0.97	2.68	3.333 (8)	125
C19—H19⋯O2^iii^	0.93	2.96	3.647 (9)	132
C20—H20⋯O5^iii^	0.93	2.88	3.788 (8)	165
C28—H28⋯O6^ii^	0.93	2.79	3.716 (9)	171
C23—H23*C*⋯*Cg*1^ii^	0.96	2.96	3.701 (3)	135
C25—H25⋯*Cg*2^iv^	0.93	2.67	3.483 (5)	147

Symmetry codes: (i) 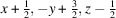; (ii) 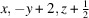; (iii) 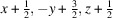; (iv) 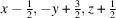.

**Table 3:** Experimental details

	**I**	**II**
Crystal data		
Chemical formula	C_24_H_22_N_2_O_4_S	C_29_H_26_N_2_O_6_S_2_
*M*_r_	434.49	562.64
Crystal system, space group	Monoclinic, *P*2_1_/*c*	Monoclinic, *Cc*
Temperature (K)	305	293
*a*, *b*, *c* (Å)	13.6698 (17), 19.781 (2), 8.1056 (10)	13.463 (9), 17.193 (12), 11.532 (7)
β (°)	99.388 (8)	94.844 (19)
*V* (Å^3^)	2162.4 (5)	2660 (3)
*Z*	4	4
Radiation type	Cu *K*α	Mo *K*α
μ (mm^−1^)	1.61	0.25
Crystal size (mm)	0.16 × 0.13 × 0.04	0.33 × 0.22 × 0.11

Data collection		
Diffractometer	Bruker D8 Venture Diffractometer	Bruker D8 Venture Diffractometer
Absorption correction	Multi-scan (*SADABS*; Krause *et al.*, 2015 ▸)	Multi-scan (*SADABS*; Krause *et al.*, 2015 ▸)
*T*_min_, *T*_max_	0.634, 0.753	0.504, 0.745
No. of measured, independent and observed [*I* > 2σ(*I*)] reflections	47234, 3963, 2595	42032, 5193, 4533
*R*_int_	0.087	0.086
(sin θ/λ)_max_ (Å^−1^)	0.604	0.628

Refinement		
*R*[*F*^2^ > 2σ(*F*^2^)], *wR*(*F*^2^), *S*	0.058, 0.179, 1.07	0.057, 0.154, 1.12
No. of reflections	3963	5193
No. of parameters	283	354
No. of restraints	0	2
H-atom treatment	H-atom parameters constrained	H-atom parameters constrained
Δρ_max_, Δρ_min_ (e Å^−3^)	0.27, −0.41	1.17, −0.26
Absolute structure	–	Flack *x* determined using 1861 quotients [(*I*^+^)−(*I*^−^)]/[(*I*^+^)+(*I*^−^)] (Parsons *et al.*, 2013 ▸)
Absolute structure parameter	–	0.16 (4)

Computer programs: *APEX2* and *SAINT* (Bruker, 2016 ▸), *SHELXS2018/3* (Sheldrick, 2008 ▸), *SHELXL2018/3* (Sheldrick, 2015 ▸), *ORTEP-3 for Windows* and *WinGX* (Farrugia, 2012 ▸), pubCIF (Westrip, 2010 ▸) and *PLATON* (Spek, 2020 ▸).

## supplementary crystallographic information

### N-(3-Methoxyphenyl)-N-{[1-(phenylsulfonyl)-1H-indol-2-yl]methyl}acetamide (I) Crystal data

**Table:**

C_24_H_22_N_2_O_4_S	*F*(000) = 912
*M**_r_* = 434.49	*D*_x_ = 1.335 Mg m^−^^3^
Monoclinic, *P*2_1_/*c*	Cu *K*α radiation, λ = 1.54178 Å
*a* = 13.6698 (17) Å	Cell parameters from 47234 reflections
*b* = 19.781 (2) Å	θ = 1.4–25.0°
*c* = 8.1056 (10) Å	µ = 1.61 mm^−^^1^
β = 99.388 (8)°	*T* = 305 K
*V* = 2162.4 (5) Å^3^	Prism, colorless
*Z* = 4	0.16 × 0.13 × 0.04 mm

### N-(3-Methoxyphenyl)-N-{[1-(phenylsulfonyl)-1H-indol-2-yl]methyl}acetamide (I) Data collection

**Table:**

Bruker D8 Venture Diffractometer	2595 reflections with *I* > 2σ(*I*)
Radiation source: micro focus sealed tube	*R*_int_ = 0.087
ω and φ scans	θ_max_ = 68.6°, θ_min_ = 3.3°
Absorption correction: multi-scan (*SADABS*; Krause *et al.*, 2015)	*h* = −16→16
*T*_min_ = 0.634, *T*_max_ = 0.753	*k* = −23→23
47234 measured reflections	*l* = −9→9
3963 independent reflections	

### N-(3-Methoxyphenyl)-N-{[1-(phenylsulfonyl)-1H-indol-2-yl]methyl}acetamide (I) Refinement

**Table:**

Refinement on *F*^2^	Secondary atom site location: difference Fourier map
Least-squares matrix: full	Hydrogen site location: inferred from neighbouring sites
*R*[*F*^2^ > 2σ(*F*^2^)] = 0.058	H-atom parameters constrained
*wR*(*F*^2^) = 0.179	*w* = 1/[σ^2^(*F*_o_^2^) + (0.0662*P*)^2^ + 2.3955*P*] where *P* = (*F*_o_^2^ + 2*F*_c_^2^)/3
*S* = 1.07	(Δ/σ)_max_ = 0.001
3963 reflections	Δρ_max_ = 0.27 e Å^−^^3^
283 parameters	Δρ_min_ = −0.41 e Å^−^^3^
0 restraints	Extinction correction: *SHELXL2018/3* (Sheldrick, 2015), Fc^*^=kFc[1+0.001xFc^2^λ^3^/sin(2θ)]^-1/4^
Primary atom site location: structure-invariant direct methods	Extinction coefficient: 0.0036 (4)

### N-(3-Methoxyphenyl)-N-{[1-(phenylsulfonyl)-1H-indol-2-yl]methyl}acetamide (I) Special details

**Table:**

Geometry. All esds (except the esd in the dihedral angle between two l.s. planes) are estimated using the full covariance matrix. The cell esds are taken into account individually in the estimation of esds in distances, angles and torsion angles; correlations between esds in cell parameters are only used when they are defined by crystal symmetry. An approximate (isotropic) treatment of cell esds is used for estimating esds involving l.s. planes.

### N-(3-Methoxyphenyl)-N-{[1-(phenylsulfonyl)-1H-indol-2-yl]methyl}acetamide (I) Fractional atomic coordinates and isotropic or equivalent isotropic displacement parameters (Å^2^)

**Table:**

	*x*	*y*	*z*	*U*_iso_*/*U*_eq_	
C1	0.5527 (2)	0.58150 (16)	0.5180 (4)	0.0494 (8)	
C2	0.4749 (3)	0.61289 (19)	0.5779 (5)	0.0613 (9)	
H2	0.471182	0.613774	0.691426	0.074*	
C3	0.4031 (3)	0.6428 (2)	0.4613 (5)	0.0727 (11)	
H3	0.350636	0.665179	0.497364	0.087*	
C4	0.4072 (3)	0.6402 (2)	0.2915 (5)	0.0781 (12)	
H4	0.357245	0.660465	0.216034	0.094*	
C5	0.4840 (3)	0.6083 (2)	0.2333 (5)	0.0673 (10)	
H5	0.485845	0.606208	0.119252	0.081*	
C6	0.5586 (2)	0.57918 (17)	0.3473 (4)	0.0513 (8)	
C7	0.6486 (3)	0.54485 (18)	0.3303 (4)	0.0555 (8)	
H7	0.670758	0.536369	0.229756	0.067*	
C8	0.6960 (2)	0.52676 (16)	0.4825 (4)	0.0502 (8)	
C9	0.7198 (3)	0.64340 (19)	0.8178 (4)	0.0599 (9)	
C10	0.6621 (4)	0.6939 (2)	0.8699 (6)	0.0867 (13)	
H10	0.600910	0.683953	0.899914	0.104*	
C11	0.6973 (5)	0.7597 (3)	0.8766 (8)	0.1113 (19)	
H11	0.659700	0.794043	0.913607	0.134*	
C12	0.7863 (5)	0.7751 (3)	0.8299 (7)	0.1083 (18)	
H12	0.808546	0.819604	0.833023	0.130*	
C13	0.8418 (4)	0.7248 (3)	0.7790 (7)	0.1038 (17)	
H13	0.902906	0.735134	0.748880	0.125*	
C14	0.8097 (3)	0.6589 (2)	0.7709 (6)	0.0824 (12)	
H14	0.848301	0.625099	0.734269	0.099*	
C15	0.7907 (3)	0.48792 (19)	0.5282 (4)	0.0591 (9)	
H15A	0.837940	0.514936	0.602804	0.071*	
H15B	0.777940	0.446945	0.586890	0.071*	
C16	0.8217 (2)	0.40106 (16)	0.3218 (4)	0.0484 (8)	
C17	0.8897 (2)	0.35387 (16)	0.3928 (4)	0.0525 (8)	
H17	0.943506	0.367152	0.471601	0.063*	
C18	0.8780 (3)	0.28657 (17)	0.3468 (4)	0.0544 (8)	
C19	0.7983 (3)	0.26690 (19)	0.2299 (5)	0.0618 (9)	
H19	0.789780	0.221723	0.199177	0.074*	
C20	0.7312 (3)	0.3150 (2)	0.1590 (5)	0.0672 (10)	
H20	0.677551	0.301742	0.079824	0.081*	
C21	0.7419 (3)	0.38218 (19)	0.2031 (4)	0.0607 (9)	
H21	0.696470	0.414191	0.153903	0.073*	
C22	0.9413 (4)	0.1736 (2)	0.3867 (6)	0.0915 (14)	
H22A	0.879674	0.156372	0.411348	0.137*	
H22B	0.995389	0.149874	0.452372	0.137*	
H22C	0.943939	0.167330	0.270103	0.137*	
C23	0.8979 (3)	0.51411 (18)	0.3257 (5)	0.0578 (9)	
C24	0.9462 (3)	0.4928 (2)	0.1812 (5)	0.0687 (10)	
H24A	0.992634	0.526852	0.159832	0.103*	
H24B	0.896515	0.487187	0.083803	0.103*	
H24C	0.980398	0.450824	0.207134	0.103*	
N1	0.63700 (19)	0.54697 (14)	0.6039 (3)	0.0495 (7)	
N2	0.8335 (2)	0.47013 (13)	0.3784 (3)	0.0516 (7)	
O1	0.5933 (2)	0.55538 (14)	0.8893 (3)	0.0732 (8)	
O2	0.7592 (2)	0.51507 (13)	0.8517 (3)	0.0735 (8)	
O3	0.9487 (2)	0.24362 (12)	0.4258 (4)	0.0755 (8)	
O4	0.9139 (2)	0.56971 (13)	0.3939 (4)	0.0815 (9)	
S1	0.67746 (7)	0.55995 (5)	0.80653 (10)	0.0577 (3)	

### N-(3-Methoxyphenyl)-N-{[1-(phenylsulfonyl)-1H-indol-2-yl]methyl}acetamide (I) Atomic displacement parameters (Å^2^)

**Table:**

	*U*^11^	*U*^22^	*U*^33^	*U*^12^	*U*^13^	*U*^23^
C1	0.0507 (18)	0.0488 (18)	0.0484 (18)	0.0017 (14)	0.0070 (14)	0.0016 (14)
C2	0.061 (2)	0.071 (2)	0.055 (2)	0.0074 (19)	0.0169 (17)	0.0000 (17)
C3	0.062 (2)	0.088 (3)	0.070 (3)	0.019 (2)	0.0174 (19)	0.004 (2)
C4	0.069 (2)	0.096 (3)	0.069 (3)	0.024 (2)	0.012 (2)	0.016 (2)
C5	0.066 (2)	0.083 (3)	0.053 (2)	0.014 (2)	0.0087 (17)	0.0099 (19)
C6	0.0535 (18)	0.0532 (19)	0.0469 (18)	0.0029 (15)	0.0074 (14)	0.0035 (14)
C7	0.062 (2)	0.062 (2)	0.0441 (18)	0.0094 (17)	0.0107 (15)	0.0019 (15)
C8	0.0541 (18)	0.0479 (18)	0.0489 (18)	0.0059 (15)	0.0094 (14)	−0.0045 (14)
C9	0.069 (2)	0.062 (2)	0.0469 (19)	0.0051 (18)	0.0015 (16)	−0.0058 (16)
C10	0.091 (3)	0.072 (3)	0.097 (3)	0.007 (2)	0.014 (3)	−0.022 (2)
C11	0.141 (5)	0.063 (3)	0.125 (5)	0.005 (3)	0.007 (4)	−0.030 (3)
C12	0.147 (5)	0.070 (3)	0.100 (4)	−0.029 (4)	−0.003 (4)	−0.009 (3)
C13	0.105 (4)	0.089 (4)	0.117 (4)	−0.033 (3)	0.018 (3)	−0.013 (3)
C14	0.078 (3)	0.078 (3)	0.093 (3)	−0.009 (2)	0.018 (2)	−0.015 (2)
C15	0.062 (2)	0.063 (2)	0.0498 (19)	0.0096 (17)	0.0032 (16)	−0.0078 (16)
C16	0.0477 (17)	0.0484 (18)	0.0498 (18)	−0.0023 (14)	0.0101 (14)	−0.0055 (14)
C17	0.0519 (18)	0.0469 (19)	0.0570 (19)	−0.0013 (15)	0.0035 (15)	−0.0051 (15)
C18	0.057 (2)	0.0495 (19)	0.057 (2)	0.0028 (16)	0.0110 (16)	0.0052 (15)
C19	0.072 (2)	0.052 (2)	0.063 (2)	−0.0132 (18)	0.0132 (18)	−0.0067 (17)
C20	0.066 (2)	0.068 (2)	0.063 (2)	−0.010 (2)	−0.0016 (18)	−0.0101 (19)
C21	0.059 (2)	0.062 (2)	0.059 (2)	0.0033 (18)	0.0037 (16)	−0.0056 (17)
C22	0.119 (4)	0.047 (2)	0.107 (4)	0.013 (2)	0.014 (3)	−0.001 (2)
C23	0.0501 (19)	0.051 (2)	0.070 (2)	0.0038 (16)	0.0001 (16)	−0.0033 (17)
C24	0.064 (2)	0.067 (2)	0.078 (3)	−0.0058 (19)	0.0183 (19)	0.005 (2)
N1	0.0545 (16)	0.0554 (16)	0.0385 (14)	0.0040 (13)	0.0077 (11)	−0.0023 (11)
N2	0.0515 (15)	0.0473 (15)	0.0566 (16)	0.0022 (13)	0.0101 (12)	−0.0067 (12)
O1	0.0866 (19)	0.0873 (19)	0.0511 (14)	−0.0085 (15)	0.0270 (13)	0.0007 (13)
O2	0.0924 (19)	0.0701 (17)	0.0512 (14)	0.0249 (15)	−0.0085 (13)	0.0035 (12)
O3	0.0819 (18)	0.0497 (15)	0.091 (2)	0.0084 (13)	0.0019 (15)	0.0007 (13)
O4	0.0768 (18)	0.0556 (16)	0.108 (2)	−0.0049 (14)	0.0034 (16)	−0.0171 (15)
S1	0.0714 (6)	0.0588 (6)	0.0420 (5)	0.0043 (4)	0.0062 (4)	−0.0007 (4)

### N-(3-Methoxyphenyl)-N-{[1-(phenylsulfonyl)-1H-indol-2-yl]methyl}acetamide (I) Geometric parameters (Å, º)

**Table:**

C1—C2	1.386 (5)	C15—N2	1.473 (4)
C1—C6	1.400 (4)	C15—H15A	0.9700
C1—N1	1.420 (4)	C15—H15B	0.9700
C2—C3	1.379 (5)	C16—C17	1.375 (5)
C2—H2	0.9300	C16—C21	1.383 (5)
C3—C4	1.387 (6)	C16—N2	1.442 (4)
C3—H3	0.9300	C17—C18	1.385 (5)
C4—C5	1.373 (5)	C17—H17	0.9300
C4—H4	0.9300	C18—O3	1.366 (4)
C5—C6	1.385 (5)	C18—C19	1.378 (5)
C5—H5	0.9300	C19—C20	1.380 (5)
C6—C7	1.431 (5)	C19—H19	0.9300
C7—C8	1.345 (4)	C20—C21	1.378 (5)
C7—H7	0.9300	C20—H20	0.9300
C8—N1	1.427 (4)	C21—H21	0.9300
C8—C15	1.499 (5)	C22—O3	1.420 (5)
C9—C14	1.380 (6)	C22—H22A	0.9600
C9—C10	1.381 (5)	C22—H22B	0.9600
C9—S1	1.746 (4)	C22—H22C	0.9600
C10—C11	1.386 (7)	C23—O4	1.234 (4)
C10—H10	0.9300	C23—N2	1.356 (4)
C11—C12	1.365 (8)	C23—C24	1.496 (5)
C11—H11	0.9300	C24—H24A	0.9600
C12—C13	1.357 (8)	C24—H24B	0.9600
C12—H12	0.9300	C24—H24C	0.9600
C13—C14	1.373 (6)	N1—S1	1.665 (3)
C13—H13	0.9300	O1—S1	1.426 (3)
C14—H14	0.9300	O2—S1	1.427 (3)
			
C2—C1—C6	122.1 (3)	H15A—C15—H15B	108.0
C2—C1—N1	130.7 (3)	C17—C16—C21	120.7 (3)
C6—C1—N1	107.2 (3)	C17—C16—N2	118.5 (3)
C3—C2—C1	116.9 (3)	C21—C16—N2	120.8 (3)
C3—C2—H2	121.5	C16—C17—C18	120.0 (3)
C1—C2—H2	121.5	C16—C17—H17	120.0
C2—C3—C4	121.7 (4)	C18—C17—H17	120.0
C2—C3—H3	119.2	O3—C18—C19	124.5 (3)
C4—C3—H3	119.2	O3—C18—C17	115.5 (3)
C5—C4—C3	121.0 (4)	C19—C18—C17	120.0 (3)
C5—C4—H4	119.5	C18—C19—C20	119.3 (3)
C3—C4—H4	119.5	C18—C19—H19	120.4
C4—C5—C6	118.9 (4)	C20—C19—H19	120.4
C4—C5—H5	120.6	C21—C20—C19	121.4 (3)
C6—C5—H5	120.6	C21—C20—H20	119.3
C5—C6—C1	119.4 (3)	C19—C20—H20	119.3
C5—C6—C7	133.1 (3)	C20—C21—C16	118.6 (3)
C1—C6—C7	107.5 (3)	C20—C21—H21	120.7
C8—C7—C6	109.3 (3)	C16—C21—H21	120.7
C8—C7—H7	125.3	O3—C22—H22A	109.5
C6—C7—H7	125.3	O3—C22—H22B	109.5
C7—C8—N1	108.4 (3)	H22A—C22—H22B	109.5
C7—C8—C15	129.1 (3)	O3—C22—H22C	109.5
N1—C8—C15	122.4 (3)	H22A—C22—H22C	109.5
C14—C9—C10	120.1 (4)	H22B—C22—H22C	109.5
C14—C9—S1	119.9 (3)	O4—C23—N2	120.5 (4)
C10—C9—S1	119.9 (3)	O4—C23—C24	122.2 (4)
C9—C10—C11	118.7 (5)	N2—C23—C24	117.3 (3)
C9—C10—H10	120.7	C23—C24—H24A	109.5
C11—C10—H10	120.7	C23—C24—H24B	109.5
C12—C11—C10	121.1 (5)	H24A—C24—H24B	109.5
C12—C11—H11	119.4	C23—C24—H24C	109.5
C10—C11—H11	119.4	H24A—C24—H24C	109.5
C13—C12—C11	119.3 (5)	H24B—C24—H24C	109.5
C13—C12—H12	120.4	C1—N1—C8	107.5 (2)
C11—C12—H12	120.4	C1—N1—S1	121.4 (2)
C12—C13—C14	121.4 (5)	C8—N1—S1	126.1 (2)
C12—C13—H13	119.3	C23—N2—C16	123.5 (3)
C14—C13—H13	119.3	C23—N2—C15	118.2 (3)
C13—C14—C9	119.4 (5)	C16—N2—C15	116.7 (3)
C13—C14—H14	120.3	C18—O3—C22	118.9 (3)
C9—C14—H14	120.3	O1—S1—O2	119.83 (17)
N2—C15—C8	111.2 (3)	O1—S1—N1	106.91 (15)
N2—C15—H15A	109.4	O2—S1—N1	106.10 (14)
C8—C15—H15A	109.4	O1—S1—C9	108.73 (18)
N2—C15—H15B	109.4	O2—S1—C9	109.62 (18)
C8—C15—H15B	109.4	N1—S1—C9	104.54 (15)
			
C6—C1—C2—C3	−0.7 (6)	N2—C16—C21—C20	−176.8 (3)
N1—C1—C2—C3	179.7 (4)	C2—C1—N1—C8	−177.6 (4)
C1—C2—C3—C4	1.5 (6)	C6—C1—N1—C8	2.7 (4)
C2—C3—C4—C5	−0.7 (7)	C2—C1—N1—S1	−21.1 (5)
C3—C4—C5—C6	−0.9 (7)	C6—C1—N1—S1	159.3 (2)
C4—C5—C6—C1	1.7 (6)	C7—C8—N1—C1	−2.7 (4)
C4—C5—C6—C7	−177.7 (4)	C15—C8—N1—C1	−179.1 (3)
C2—C1—C6—C5	−0.9 (5)	C7—C8—N1—S1	−157.9 (3)
N1—C1—C6—C5	178.8 (3)	C15—C8—N1—S1	25.7 (5)
C2—C1—C6—C7	178.6 (3)	O4—C23—N2—C16	−170.7 (3)
N1—C1—C6—C7	−1.7 (4)	C24—C23—N2—C16	10.4 (5)
C5—C6—C7—C8	179.4 (4)	O4—C23—N2—C15	−5.6 (5)
C1—C6—C7—C8	0.0 (4)	C24—C23—N2—C15	175.6 (3)
C6—C7—C8—N1	1.7 (4)	C17—C16—N2—C23	80.1 (4)
C6—C7—C8—C15	177.7 (3)	C21—C16—N2—C23	−102.1 (4)
C14—C9—C10—C11	−1.1 (7)	C17—C16—N2—C15	−85.2 (4)
S1—C9—C10—C11	−179.6 (4)	C21—C16—N2—C15	92.5 (4)
C9—C10—C11—C12	1.2 (8)	C8—C15—N2—C23	90.1 (4)
C10—C11—C12—C13	−1.2 (9)	C8—C15—N2—C16	−103.7 (3)
C11—C12—C13—C14	1.0 (9)	C19—C18—O3—C22	0.1 (6)
C12—C13—C14—C9	−0.9 (8)	C17—C18—O3—C22	179.4 (4)
C10—C9—C14—C13	0.9 (7)	C1—N1—S1—O1	48.3 (3)
S1—C9—C14—C13	179.5 (4)	C8—N1—S1—O1	−159.7 (3)
C7—C8—C15—N2	1.1 (5)	C1—N1—S1—O2	177.3 (3)
N1—C8—C15—N2	176.6 (3)	C8—N1—S1—O2	−30.7 (3)
C21—C16—C17—C18	−0.7 (5)	C1—N1—S1—C9	−66.9 (3)
N2—C16—C17—C18	177.0 (3)	C8—N1—S1—C9	85.1 (3)
C16—C17—C18—O3	−179.3 (3)	C14—C9—S1—O1	169.5 (3)
C16—C17—C18—C19	0.1 (5)	C10—C9—S1—O1	−11.9 (4)
O3—C18—C19—C20	179.7 (3)	C14—C9—S1—O2	36.8 (4)
C17—C18—C19—C20	0.4 (5)	C10—C9—S1—O2	−144.7 (3)
C18—C19—C20—C21	−0.3 (6)	C14—C9—S1—N1	−76.6 (3)
C19—C20—C21—C16	−0.4 (6)	C10—C9—S1—N1	102.0 (3)
C17—C16—C21—C20	0.9 (5)		

### N-(3-Methoxyphenyl)-N-{[1-(phenylsulfonyl)-1H-indol-2-yl]methyl}acetamide (I) Hydrogen-bond geometry (Å, º)

**Table:**

*D*—H···*A*	*D*—H	H···*A*	*D*···*A*	*D*—H···*A*
C2—H2···O1	0.93	2.41	2.993 (5)	120
C5—H5···O1^i^	0.93	2.75	3.530 (5)	143
C7—H7···O1^i^	0.93	2.81	3.537 (4)	135
C12—H12···O4^ii^	0.93	2.62	3.527 (6)	164
C13—H13···O3^iii^	0.93	2.69	3.591 (7)	164
C17—H17···O4^iii^	0.93	2.42	3.312 (4)	161
C24—H24*B*···O2^i^	0.96	2.49	3.410 (5)	160

Symmetry codes: (i) *x*, *y*, *z*−1; (ii) *x*, −*y*+3/2, *z*+1/2; (iii) −*x*+2, −*y*+1, −*z*+1.

### N-(2,5-Dimethoxyphenyl)-N-{[1-(phenylsulfonyl)-1H-indol-2-yl]methyl}benzenesulfonamide (II) Crystal data

**Table:**

C_29_H_26_N_2_O_6_S_2_	*F*(000) = 1176
*M**_r_* = 562.64	*D*_x_ = 1.405 Mg m^−^^3^
Monoclinic, *C**c*	Mo *K*α radiation, λ = 0.71073 Å
*a* = 13.463 (9) Å	Cell parameters from 42032 reflections
*b* = 17.193 (12) Å	θ = 1.4–25.0°
*c* = 11.532 (7) Å	µ = 0.25 mm^−^^1^
β = 94.844 (19)°	*T* = 293 K
*V* = 2660 (3) Å^3^	Prism, colorless
*Z* = 4	0.33 × 0.22 × 0.11 mm

### N-(2,5-Dimethoxyphenyl)-N-{[1-(phenylsulfonyl)-1H-indol-2-yl]methyl}benzenesulfonamide (II) Data collection

**Table:**

Bruker D8 Venture Diffractometer	4533 reflections with *I* > 2σ(*I*)
Radiation source: micro focus sealed tube	*R*_int_ = 0.086
ω and φ scans	θ_max_ = 26.5°, θ_min_ = 3.6°
Absorption correction: multi-scan (*SADABS*; Krause *et al.*, 2015)	*h* = −16→16
*T*_min_ = 0.504, *T*_max_ = 0.745	*k* = −21→21
42032 measured reflections	*l* = −14→14
5193 independent reflections	

### N-(2,5-Dimethoxyphenyl)-N-{[1-(phenylsulfonyl)-1H-indol-2-yl]methyl}benzenesulfonamide (II) Refinement

**Table:**

Refinement on *F*^2^	Secondary atom site location: difference Fourier map
Least-squares matrix: full	Hydrogen site location: inferred from neighbouring sites
*R*[*F*^2^ > 2σ(*F*^2^)] = 0.057	H-atom parameters constrained
*wR*(*F*^2^) = 0.154	*w* = 1/[σ^2^(*F*_o_^2^) + (0.0731*P*)^2^ + 3.4858*P*] where *P* = (*F*_o_^2^ + 2*F*_c_^2^)/3
*S* = 1.12	(Δ/σ)_max_ < 0.001
5193 reflections	Δρ_max_ = 1.17 e Å^−^^3^
354 parameters	Δρ_min_ = −0.26 e Å^−^^3^
2 restraints	Absolute structure: Flack *x* determined using 1861 quotients [(*I*^+^)-(*I*^-^)]/[(*I*^+^)+(*I*^-^)] (Parsons *et al.*, 2013)
Primary atom site location: structure-invariant direct methods	Absolute structure parameter: 0.16 (4)

### N-(2,5-Dimethoxyphenyl)-N-{[1-(phenylsulfonyl)-1H-indol-2-yl]methyl}benzenesulfonamide (II) Special details

**Table:**

Geometry. All esds (except the esd in the dihedral angle between two l.s. planes) are estimated using the full covariance matrix. The cell esds are taken into account individually in the estimation of esds in distances, angles and torsion angles; correlations between esds in cell parameters are only used when they are defined by crystal symmetry. An approximate (isotropic) treatment of cell esds is used for estimating esds involving l.s. planes.

### N-(2,5-Dimethoxyphenyl)-N-{[1-(phenylsulfonyl)-1H-indol-2-yl]methyl}benzenesulfonamide (II) Fractional atomic coordinates and isotropic or equivalent isotropic displacement parameters (Å^2^)

**Table:**

	*x*	*y*	*z*	*U*_iso_*/*U*_eq_	
C1	0.5561 (5)	0.9136 (3)	0.2353 (5)	0.0405 (13)	
C2	0.5964 (6)	0.9217 (4)	0.1289 (6)	0.0540 (16)	
H2	0.555973	0.922012	0.059422	0.065*	
C3	0.6984 (6)	0.9292 (4)	0.1301 (7)	0.0629 (19)	
H3	0.727709	0.933603	0.060166	0.076*	
C4	0.7585 (6)	0.9304 (5)	0.2352 (8)	0.068 (2)	
H4	0.827185	0.935784	0.234498	0.081*	
C5	0.7173 (5)	0.9237 (5)	0.3382 (8)	0.0611 (18)	
H5	0.758088	0.924447	0.407445	0.073*	
C6	0.6143 (5)	0.9157 (3)	0.3416 (6)	0.0457 (14)	
C7	0.5506 (5)	0.9086 (4)	0.4325 (6)	0.0474 (15)	
H7	0.571262	0.908568	0.511531	0.057*	
C8	0.4548 (4)	0.9019 (3)	0.3871 (5)	0.0368 (12)	
C9	0.3602 (5)	0.7823 (3)	0.1536 (5)	0.0419 (13)	
C10	0.4339 (5)	0.7464 (4)	0.0981 (7)	0.0556 (17)	
H10	0.484497	0.775373	0.068826	0.067*	
C11	0.4316 (6)	0.6650 (4)	0.0862 (9)	0.070 (2)	
H11	0.481400	0.639367	0.050114	0.084*	
C12	0.3561 (7)	0.6243 (5)	0.1277 (8)	0.071 (2)	
H12	0.354691	0.570506	0.119685	0.085*	
C13	0.2819 (7)	0.6605 (5)	0.1812 (8)	0.074 (2)	
H13	0.230384	0.631258	0.207941	0.088*	
C14	0.2831 (6)	0.7404 (5)	0.1957 (6)	0.0608 (19)	
H14	0.233290	0.765304	0.232769	0.073*	
C15	0.3647 (5)	0.9029 (3)	0.4548 (5)	0.0400 (12)	
H15A	0.381098	0.928997	0.528504	0.048*	
H15B	0.312174	0.932270	0.411961	0.048*	
C16	0.3950 (4)	0.7701 (3)	0.5439 (5)	0.0371 (12)	
C17	0.4070 (5)	0.6956 (4)	0.4999 (5)	0.0443 (13)	
H17	0.375102	0.681947	0.428127	0.053*	
C18	0.4660 (5)	0.6420 (4)	0.5620 (6)	0.0481 (15)	
C19	0.5150 (5)	0.6612 (4)	0.6672 (6)	0.0552 (17)	
H19	0.555292	0.624805	0.707987	0.066*	
C20	0.5043 (5)	0.7351 (4)	0.7125 (6)	0.0531 (16)	
H20	0.537510	0.748217	0.783811	0.064*	
C21	0.4437 (4)	0.7899 (4)	0.6516 (5)	0.0422 (13)	
C22	0.4410 (7)	0.5443 (5)	0.4146 (8)	0.074 (2)	
H22A	0.461365	0.581454	0.359113	0.110*	
H22B	0.369542	0.542977	0.411544	0.110*	
H22C	0.465569	0.493729	0.396292	0.110*	
C23	0.4596 (8)	0.8823 (5)	0.8082 (7)	0.077 (2)	
H23A	0.531027	0.880524	0.820273	0.115*	
H23B	0.431424	0.845285	0.858364	0.115*	
H23C	0.436809	0.933578	0.825521	0.115*	
C24	0.1965 (4)	0.8362 (4)	0.6486 (5)	0.0415 (13)	
C25	0.1984 (5)	0.7775 (4)	0.7286 (6)	0.0531 (16)	
H25	0.206152	0.726136	0.705626	0.064*	
C26	0.1887 (6)	0.7953 (5)	0.8457 (7)	0.067 (2)	
H26	0.189399	0.755573	0.900444	0.081*	
C27	0.1782 (6)	0.8710 (5)	0.8800 (7)	0.065 (2)	
H27	0.172197	0.882626	0.957870	0.078*	
C28	0.1766 (6)	0.9298 (5)	0.7985 (7)	0.065 (2)	
H28	0.170116	0.981104	0.822519	0.077*	
C29	0.1843 (5)	0.9143 (4)	0.6818 (7)	0.0540 (16)	
H29	0.181569	0.954118	0.627016	0.065*	
N1	0.4553 (4)	0.9058 (3)	0.2641 (4)	0.0389 (10)	
N2	0.3279 (4)	0.8234 (3)	0.4772 (4)	0.0363 (10)	
O1	0.3855 (4)	0.9166 (3)	0.0595 (4)	0.0592 (12)	
O2	0.2718 (4)	0.9068 (3)	0.2140 (4)	0.0608 (13)	
O3	0.4797 (5)	0.5658 (3)	0.5265 (5)	0.0713 (15)	
O4	0.4295 (4)	0.8639 (3)	0.6904 (4)	0.0544 (11)	
O5	0.1821 (3)	0.7351 (3)	0.4813 (4)	0.0556 (12)	
O6	0.1575 (3)	0.8740 (3)	0.4303 (5)	0.0573 (12)	
S1	0.36074 (11)	0.88404 (9)	0.16552 (11)	0.0423 (4)	
S2	0.20853 (11)	0.81497 (9)	0.50035 (12)	0.0414 (4)	

### N-(2,5-Dimethoxyphenyl)-N-{[1-(phenylsulfonyl)-1H-indol-2-yl]methyl}benzenesulfonamide (II) Atomic displacement parameters (Å^2^)

**Table:**

	*U*^11^	*U*^22^	*U*^33^	*U*^12^	*U*^13^	*U*^23^
C1	0.050 (3)	0.031 (3)	0.041 (3)	−0.009 (2)	0.010 (3)	0.000 (2)
C2	0.062 (4)	0.051 (4)	0.050 (4)	−0.011 (3)	0.011 (3)	−0.004 (3)
C3	0.070 (5)	0.057 (4)	0.065 (5)	−0.014 (4)	0.030 (4)	0.002 (3)
C4	0.053 (4)	0.063 (5)	0.089 (6)	−0.016 (3)	0.017 (4)	−0.007 (4)
C5	0.044 (3)	0.068 (5)	0.072 (5)	−0.011 (3)	0.004 (3)	0.003 (4)
C6	0.051 (3)	0.035 (3)	0.050 (4)	−0.005 (3)	−0.003 (3)	0.000 (2)
C7	0.048 (3)	0.052 (4)	0.041 (3)	−0.010 (3)	−0.004 (3)	0.003 (3)
C8	0.047 (3)	0.032 (3)	0.031 (3)	−0.005 (2)	0.002 (2)	−0.001 (2)
C9	0.042 (3)	0.041 (3)	0.040 (3)	−0.001 (3)	−0.010 (2)	−0.002 (3)
C10	0.043 (3)	0.050 (4)	0.074 (5)	−0.003 (3)	0.002 (3)	−0.011 (3)
C11	0.053 (4)	0.051 (4)	0.104 (7)	0.003 (3)	−0.008 (4)	−0.019 (4)
C12	0.086 (6)	0.050 (4)	0.074 (5)	−0.012 (4)	−0.011 (5)	−0.003 (4)
C13	0.091 (6)	0.067 (5)	0.064 (5)	−0.036 (5)	0.011 (4)	−0.003 (4)
C14	0.056 (4)	0.074 (5)	0.052 (4)	−0.007 (4)	0.003 (3)	−0.015 (3)
C15	0.049 (3)	0.035 (3)	0.036 (3)	0.001 (2)	0.003 (2)	−0.003 (2)
C16	0.039 (3)	0.041 (3)	0.032 (3)	−0.001 (2)	0.003 (2)	−0.003 (2)
C17	0.044 (3)	0.047 (3)	0.040 (3)	−0.006 (3)	−0.004 (2)	−0.003 (2)
C18	0.043 (3)	0.038 (3)	0.062 (4)	−0.003 (3)	0.002 (3)	0.003 (3)
C19	0.046 (4)	0.063 (4)	0.055 (4)	0.006 (3)	−0.007 (3)	0.013 (3)
C20	0.044 (3)	0.066 (4)	0.047 (4)	−0.003 (3)	−0.011 (3)	−0.001 (3)
C21	0.038 (3)	0.050 (3)	0.037 (3)	−0.003 (3)	0.000 (2)	−0.005 (2)
C22	0.071 (5)	0.056 (5)	0.092 (6)	−0.006 (4)	−0.005 (4)	−0.023 (4)
C23	0.104 (7)	0.079 (6)	0.044 (4)	0.005 (5)	−0.013 (4)	−0.018 (4)
C24	0.035 (3)	0.049 (3)	0.041 (3)	−0.006 (2)	0.006 (2)	−0.007 (2)
C25	0.062 (4)	0.049 (4)	0.049 (4)	−0.002 (3)	0.006 (3)	0.002 (3)
C26	0.078 (5)	0.080 (5)	0.044 (4)	−0.006 (4)	0.007 (4)	−0.001 (4)
C27	0.059 (4)	0.089 (6)	0.049 (4)	−0.008 (4)	0.010 (3)	−0.020 (4)
C28	0.060 (4)	0.064 (5)	0.071 (5)	−0.015 (4)	0.017 (4)	−0.027 (4)
C29	0.057 (4)	0.047 (3)	0.059 (4)	−0.012 (3)	0.015 (3)	−0.008 (3)
N1	0.042 (3)	0.042 (3)	0.032 (2)	−0.004 (2)	0.0000 (19)	−0.0013 (19)
N2	0.037 (2)	0.039 (2)	0.033 (2)	−0.0031 (19)	0.0025 (19)	0.0005 (18)
O1	0.083 (3)	0.055 (3)	0.037 (2)	0.001 (2)	−0.008 (2)	−0.002 (2)
O2	0.052 (3)	0.073 (3)	0.055 (3)	0.014 (2)	−0.009 (2)	−0.014 (2)
O3	0.084 (4)	0.048 (3)	0.081 (4)	0.010 (3)	−0.005 (3)	−0.003 (3)
O4	0.067 (3)	0.056 (3)	0.038 (2)	−0.003 (2)	−0.007 (2)	−0.0134 (19)
O5	0.055 (3)	0.065 (3)	0.045 (3)	−0.018 (2)	−0.001 (2)	−0.011 (2)
O6	0.046 (2)	0.069 (3)	0.055 (3)	0.007 (2)	−0.005 (2)	0.010 (2)
S1	0.0484 (8)	0.0465 (7)	0.0305 (7)	0.0067 (7)	−0.0057 (6)	−0.0013 (6)
S2	0.0377 (7)	0.0507 (8)	0.0350 (7)	−0.0053 (6)	−0.0013 (5)	−0.0019 (6)

### N-(2,5-Dimethoxyphenyl)-N-{[1-(phenylsulfonyl)-1H-indol-2-yl]methyl}benzenesulfonamide (II) Geometric parameters (Å, º)

**Table:**

C1—C2	1.390 (9)	C17—H17	0.9300
C1—C6	1.398 (9)	C18—C19	1.371 (10)
C1—N1	1.429 (8)	C18—O3	1.389 (8)
C2—C3	1.379 (11)	C19—C20	1.385 (10)
C2—H2	0.9300	C19—H19	0.9300
C3—C4	1.399 (13)	C20—C21	1.396 (9)
C3—H3	0.9300	C20—H20	0.9300
C4—C5	1.358 (12)	C21—O4	1.368 (8)
C4—H4	0.9300	C22—O3	1.400 (10)
C5—C6	1.397 (10)	C22—H22A	0.9600
C5—H5	0.9300	C22—H22B	0.9600
C6—C7	1.416 (10)	C22—H22C	0.9600
C7—C8	1.355 (9)	C23—O4	1.420 (8)
C7—H7	0.9300	C23—H23A	0.9600
C8—N1	1.421 (7)	C23—H23B	0.9600
C8—C15	1.497 (8)	C23—H23C	0.9600
C9—C10	1.372 (10)	C24—C25	1.367 (9)
C9—C14	1.385 (10)	C24—C29	1.410 (9)
C9—S1	1.755 (6)	C24—S2	1.768 (6)
C10—C11	1.406 (10)	C25—C26	1.401 (10)
C10—H10	0.9300	C25—H25	0.9300
C11—C12	1.354 (13)	C26—C27	1.371 (12)
C11—H11	0.9300	C26—H26	0.9300
C12—C13	1.367 (13)	C27—C28	1.380 (13)
C12—H12	0.9300	C27—H27	0.9300
C13—C14	1.383 (12)	C28—C29	1.385 (11)
C13—H13	0.9300	C28—H28	0.9300
C14—H14	0.9300	C29—H29	0.9300
C15—N2	1.484 (7)	N1—S1	1.676 (5)
C15—H15A	0.9700	N2—S2	1.658 (5)
C15—H15B	0.9700	O1—S1	1.410 (5)
C16—C17	1.392 (8)	O2—S1	1.419 (5)
C16—C21	1.397 (8)	O5—S2	1.431 (5)
C16—N2	1.459 (7)	O6—S2	1.436 (5)
C17—C18	1.377 (9)		
			
C2—C1—C6	122.6 (6)	C18—C19—H19	120.1
C2—C1—N1	131.5 (6)	C20—C19—H19	120.1
C6—C1—N1	105.8 (5)	C19—C20—C21	120.2 (6)
C3—C2—C1	117.6 (7)	C19—C20—H20	119.9
C3—C2—H2	121.2	C21—C20—H20	119.9
C1—C2—H2	121.2	O4—C21—C20	123.7 (5)
C2—C3—C4	120.8 (7)	O4—C21—C16	116.7 (5)
C2—C3—H3	119.6	C20—C21—C16	119.6 (6)
C4—C3—H3	119.6	O3—C22—H22A	109.5
C5—C4—C3	120.5 (7)	O3—C22—H22B	109.5
C5—C4—H4	119.7	H22A—C22—H22B	109.5
C3—C4—H4	119.7	O3—C22—H22C	109.5
C4—C5—C6	120.8 (8)	H22A—C22—H22C	109.5
C4—C5—H5	119.6	H22B—C22—H22C	109.5
C6—C5—H5	119.6	O4—C23—H23A	109.5
C5—C6—C1	117.6 (7)	O4—C23—H23B	109.5
C5—C6—C7	134.0 (7)	H23A—C23—H23B	109.5
C1—C6—C7	108.5 (6)	O4—C23—H23C	109.5
C8—C7—C6	109.7 (6)	H23A—C23—H23C	109.5
C8—C7—H7	125.1	H23B—C23—H23C	109.5
C6—C7—H7	125.1	C25—C24—C29	121.1 (6)
C7—C8—N1	107.3 (5)	C25—C24—S2	120.2 (5)
C7—C8—C15	125.7 (5)	C29—C24—S2	118.8 (5)
N1—C8—C15	126.4 (5)	C24—C25—C26	119.4 (7)
C10—C9—C14	121.5 (6)	C24—C25—H25	120.3
C10—C9—S1	119.1 (5)	C26—C25—H25	120.3
C14—C9—S1	119.3 (6)	C27—C26—C25	120.4 (8)
C9—C10—C11	118.8 (7)	C27—C26—H26	119.8
C9—C10—H10	120.6	C25—C26—H26	119.8
C11—C10—H10	120.6	C26—C27—C28	119.7 (7)
C12—C11—C10	119.4 (8)	C26—C27—H27	120.2
C12—C11—H11	120.3	C28—C27—H27	120.2
C10—C11—H11	120.3	C27—C28—C29	121.5 (7)
C11—C12—C13	121.6 (7)	C27—C28—H28	119.3
C11—C12—H12	119.2	C29—C28—H28	119.3
C13—C12—H12	119.2	C28—C29—C24	117.9 (7)
C12—C13—C14	120.3 (8)	C28—C29—H29	121.1
C12—C13—H13	119.8	C24—C29—H29	121.1
C14—C13—H13	119.8	C8—N1—C1	108.7 (5)
C13—C14—C9	118.4 (7)	C8—N1—S1	126.8 (4)
C13—C14—H14	120.8	C1—N1—S1	123.0 (4)
C9—C14—H14	120.8	C16—N2—C15	118.0 (5)
N2—C15—C8	112.2 (5)	C16—N2—S2	115.2 (4)
N2—C15—H15A	109.2	C15—N2—S2	116.8 (4)
C8—C15—H15A	109.2	C18—O3—C22	118.2 (6)
N2—C15—H15B	109.2	C21—O4—C23	119.0 (6)
C8—C15—H15B	109.2	O1—S1—O2	120.1 (3)
H15A—C15—H15B	107.9	O1—S1—N1	106.1 (3)
C17—C16—C21	119.2 (5)	O2—S1—N1	106.8 (3)
C17—C16—N2	118.1 (5)	O1—S1—C9	109.1 (3)
C21—C16—N2	122.6 (5)	O2—S1—C9	107.9 (3)
C18—C17—C16	120.4 (6)	N1—S1—C9	105.9 (3)
C18—C17—H17	119.8	O5—S2—O6	119.4 (3)
C16—C17—H17	119.8	O5—S2—N2	106.9 (3)
C19—C18—C17	120.8 (6)	O6—S2—N2	105.8 (3)
C19—C18—O3	115.0 (6)	O5—S2—C24	107.7 (3)
C17—C18—O3	124.2 (6)	O6—S2—C24	108.6 (3)
C18—C19—C20	119.8 (6)	N2—S2—C24	107.9 (3)
			
C6—C1—C2—C3	2.4 (10)	C25—C24—C29—C28	1.4 (10)
N1—C1—C2—C3	179.0 (6)	S2—C24—C29—C28	−179.0 (6)
C1—C2—C3—C4	−1.4 (11)	C7—C8—N1—C1	1.2 (6)
C2—C3—C4—C5	0.3 (12)	C15—C8—N1—C1	172.4 (5)
C3—C4—C5—C6	−0.1 (12)	C7—C8—N1—S1	167.7 (4)
C4—C5—C6—C1	1.0 (10)	C15—C8—N1—S1	−21.2 (8)
C4—C5—C6—C7	−179.1 (8)	C2—C1—N1—C8	−178.1 (6)
C2—C1—C6—C5	−2.2 (9)	C6—C1—N1—C8	−1.1 (6)
N1—C1—C6—C5	−179.6 (6)	C2—C1—N1—S1	14.8 (9)
C2—C1—C6—C7	177.9 (6)	C6—C1—N1—S1	−168.1 (4)
N1—C1—C6—C7	0.5 (6)	C17—C16—N2—C15	−131.1 (6)
C5—C6—C7—C8	−179.7 (7)	C21—C16—N2—C15	51.7 (7)
C1—C6—C7—C8	0.2 (7)	C17—C16—N2—S2	84.4 (6)
C6—C7—C8—N1	−0.9 (7)	C21—C16—N2—S2	−92.8 (6)
C6—C7—C8—C15	−172.1 (5)	C8—C15—N2—C16	60.6 (6)
C14—C9—C10—C11	−1.3 (11)	C8—C15—N2—S2	−155.5 (4)
S1—C9—C10—C11	−178.4 (6)	C19—C18—O3—C22	−173.2 (7)
C9—C10—C11—C12	1.2 (12)	C17—C18—O3—C22	7.9 (10)
C10—C11—C12—C13	0.0 (13)	C20—C21—O4—C23	−13.8 (10)
C11—C12—C13—C14	−0.9 (13)	C16—C21—O4—C23	167.1 (7)
C12—C13—C14—C9	0.8 (12)	C8—N1—S1—O1	164.0 (5)
C10—C9—C14—C13	0.4 (11)	C1—N1—S1—O1	−31.3 (5)
S1—C9—C14—C13	177.4 (6)	C8—N1—S1—O2	34.8 (5)
C7—C8—C15—N2	−98.6 (7)	C1—N1—S1—O2	−160.5 (5)
N1—C8—C15—N2	91.8 (6)	C8—N1—S1—C9	−80.1 (5)
C21—C16—C17—C18	0.2 (9)	C1—N1—S1—C9	84.6 (5)
N2—C16—C17—C18	−177.1 (5)	C10—C9—S1—O1	41.5 (6)
C16—C17—C18—C19	−1.0 (10)	C14—C9—S1—O1	−135.6 (5)
C16—C17—C18—O3	177.9 (6)	C10—C9—S1—O2	173.5 (5)
C17—C18—C19—C20	0.8 (10)	C14—C9—S1—O2	−3.6 (6)
O3—C18—C19—C20	−178.2 (7)	C10—C9—S1—N1	−72.4 (6)
C18—C19—C20—C21	0.1 (11)	C14—C9—S1—N1	110.5 (5)
C19—C20—C21—O4	−179.9 (6)	C16—N2—S2—O5	−53.8 (4)
C19—C20—C21—C16	−0.8 (10)	C15—N2—S2—O5	161.2 (4)
C17—C16—C21—O4	179.8 (5)	C16—N2—S2—O6	177.9 (4)
N2—C16—C21—O4	−3.0 (8)	C15—N2—S2—O6	32.9 (5)
C17—C16—C21—C20	0.6 (9)	C16—N2—S2—C24	61.8 (5)
N2—C16—C21—C20	177.8 (5)	C15—N2—S2—C24	−83.2 (5)
C29—C24—C25—C26	−0.3 (10)	C25—C24—S2—O5	21.3 (6)
S2—C24—C25—C26	−180.0 (6)	C29—C24—S2—O5	−158.4 (5)
C24—C25—C26—C27	−0.6 (12)	C25—C24—S2—O6	151.9 (5)
C25—C26—C27—C28	0.4 (12)	C29—C24—S2—O6	−27.7 (6)
C26—C27—C28—C29	0.7 (12)	C25—C24—S2—N2	−93.8 (5)
C27—C28—C29—C24	−1.5 (11)	C29—C24—S2—N2	86.5 (5)

### N-(2,5-Dimethoxyphenyl)-N-{[1-(phenylsulfonyl)-1H-indol-2-yl]methyl}benzenesulfonamide (II) Hydrogen-bond geometry (Å, º)

*Cg*1 and *Cg*2 are the centroids of the N1/C1/C6–C8 and C1–C6 rings, respectively.

**Table:**

*D*—H···*A*	*D*—H	H···*A*	*D*···*A*	*D*—H···*A*
C2—H2···O1	0.93	2.30	2.886 (9)	121
C15—H15*A*···O4	0.97	2.23	2.862 (8)	122
C15—H15*B*···O2	0.97	2.34	2.948 (8)	120
C10—H10···O5^i^	0.93	2.93	3.719 (9)	144
C11—H11···O6^i^	0.93	2.85	3.723 (11)	156
C15—H15*A*···O1^ii^	0.97	2.68	3.333 (8)	125
C19—H19···O2^iii^	0.93	2.96	3.647 (9)	132
C20—H20···O5^iii^	0.93	2.88	3.788 (8)	165
C28—H28···O6^ii^	0.93	2.79	3.716 (9)	171
C23—H23*C*···*Cg*1^ii^	0.96	2.96	3.701 (3)	135
C25—H25···*Cg*2^iv^	0.93	2.67	3.483 (5)	147

Symmetry codes: (i) *x*+1/2, −*y*+3/2, *z*−1/2; (ii) *x*, −*y*+2, *z*+1/2; (iii) *x*+1/2, −*y*+3/2, *z*+1/2; (iv) *x*−1/2, −*y*+3/2, *z*+1/2.
